# Genomic Scan Reveals Loci under Altitude Adaptation in Tibetan and Dahe Pigs

**DOI:** 10.1371/journal.pone.0110520

**Published:** 2014-10-17

**Authors:** Kunzhe Dong, Na Yao, Yabin Pu, Xiaohong He, Qianjun Zhao, Yizhao Luan, Weijun Guan, Shaoqi Rao, Yuehui Ma

**Affiliations:** 1 Institute of Animal Science, Chinese Academy of Agricultural Sciences, Beijing, China; 2 Institute for Medical Systems Biology and Department of Medical Statistics and Epidemiology, Guangdong Medical College, Dongguan, China; Wageningen UR Livestock Research, Netherlands

## Abstract

High altitude environments are of particular interest in the studies of local adaptation as well as their implications in physiology and clinical medicine in human. Some Chinese pig breeds, such as Tibetan pig (TBP) that is well adapted to the high altitude and Dahe pig (DHP) that dwells at the moderate altitude, provide ideal materials to study local adaptation to altitudes. Yet, it is still short of in-depth analysis and understanding of the genetic adaptation to high altitude in the two pig populations. In this study we conducted a genomic scan for selective sweeps using *F_ST_* to identify genes showing evidence of local adaptations in TBP and DHP, with Wuzhishan pig (WZSP) as the low-altitude reference. Totally, we identified 12 specific selective genes (*CCBE1, F2RL1, AGGF1, ZFPM2, IL2, FGF5, PLA2G4A, ADAMTS9, NRBF2, JMJD1C*, *VEGFC* and *ADAM19*) for TBP and six (*OGG1*, *FOXM*, *FLT3*, *RTEL1*, *CRELD1* and *RHOG*) for DHP. In addition, six selective genes (*VPS13A*, *GNA14, GDAP1, PARP8, FGF10 and ADAMTS16*) were shared by the two pig breeds. Among these selective genes, three (*VEGFC*, *FGF10* and *ADAMTS9*) were previously reported to be linked to the local adaptation to high altitudes in pigs, while many others were newly identified by this study. Further bioinformatics analysis demonstrated that majority of these selective signatures have some biological functions relevant to the altitude adaptation, for examples, response to hypoxia, development of blood vessels, DNA repair and several hematological involvements. These results suggest that the local adaptation to high altitude environments is sophisticated, involving numerous genes and multiple biological processes, and the shared selective signatures by the two pig breeds may provide an effective avenue to identify the common adaptive mechanisms to different altitudes.

## Introduction

Qinghai-Tibetan plateau, the highest region on the world, offers an ideal nature laboratory for studying adaptation to extreme environments. It is well known that many mammalian species living on this plateau have acquired various anatomical and physiological traits that contribute to their ability to survive in high mountains or plateau, including loss of hypoxia pulmonary vasoconstriction, right ventricle hypertrophy, thinner-walled pulmonary arterioles, high blood flow, large lungs and hearts, and high energy metabolism [1]. All the above adaptive features are considered having a genetic basis. In the past few years, increasing attentions have been devoted to identify the underlying genetic factors for Tibetan people [2], [3], yak [4], Tibetan chicken [5], Tibetan antelope [6], pika [7] as well as Tibetan pigs [8], [9]. A list of selective genes possibly responsible for high-altitude adaptation was identified for each species. However, even for the most studied Tibetan people, we lack adequate evidence from independent studies demonstrating their truly roles of these genes in the local adaptation to high altitudes.

Pig has many similarities to human at the levels of anatomy, physiology and genetics. Therefore, understanding the genetic basis of pigs for adaptation to the high altitude environments would provide vital information for human traits. However, compared to Tibetan people, the pig residents on this plateau were less studied. Two recent population genetics studies [8], [9] have proposed that a set of genes are responsible for adaptation to high altitudes in TBP. In addition, an previous experimental study revealed that several physiological measures, including pulmonary artery pressure, pulmonary artery wedge pressure, CO cardiac output and pulmonary vascular resistance in pigs, increased progressively with the simulated conditions corresponding to altitudes of 0 (low-altitude), 2300 (moderate-altitude) and 4500 meters (high-altitude) [10]. Yet, our understanding of the genetic basis underlying the adaptation process to hypoxia is still very limited, and several fundamental questions remain unresolved. For instances, it is still not very clear if moderate altitude could trigger the genetic adaptation in pigs, and whether pigs living in different level of altitude have the same or similar genetic adaptive mechanism(s) to the unfavorable harsh environment. Two local pig breeds living in South-Western China, Tibetan pig (TBP) and Dahe pig (DHP), provide golden opportunities to obtain the preliminary results pertaining to these issues. TBP, surviving in a wild state of a national natural reservation zone at the altitude of above 2500 meters, is deemed to have a local origin on Qinghai-Tibetan Plateau and has evolved exceptional mechanisms to favor their survival under high-altitude environment [11]. DHP is a native pig breed originating from a limited region in Yunnan province. Its habitat is at an altitude of about 2000 meters with oxygen pressure decreasing to ∼70% of that at sea level [12].

To carefully examine the genetic adaptation to different levels of altitude in TBP and DHP, we conducted two genomic scans for selective sweeps using *F_ST_* based on genetic differentiation, compared with WZSP, a low-land breed living in the low altitude environment in Hainan province. Similar to TBP, WZSP is raised in a flexible diet system and adapts well to grazing conditions of the forests and pastures [13]. Hence, WZSP is expected to have less artificial selection. This feature may help us to easily identify the loci shaped by natural selection, in particular, the selection signatures due to various environmental factors at high altitudes.

## Materials and Methods

### Ethics statement

This study was approved by the ethics committees of all the participating institutes (Institute of Animal Science, Chinese Academy of Agricultural Sciences, and Guangdong Medical College). All the animal experimental procedures were performed according to the guidelines for the care and use of experimental animals established by the Ministry of Agriculture of People’s Republic of China.

### Samples

A total of 96 pigs were randomly sampled from three geographic regions. TBP sample (*n* = 35) came from Nyingchi Prefecture in the Tibetan Autonomous Region of China, with an average altitude of 3000 meters. DHP sample (*n* = 27) was collected from Dahe Town in Fuyuan county of Yunnan province, which is situated at a moderate altitude (from 1700 to 2341 meters above the sea level). WZSP sample (*n* = 34) was collected from Hainan province with an average altitude of 200 meters. In an effort to avoid relatedness between the collected pigs, we consulted with the local technicians or farmers regarding their distribution and breeding history. Genomic DNA of all samples was extracted from blood according to the standard protocols provided by the manufactures.

### Genotyping and data quality control

DNA samples were genotyped using the PorcineSNP60 chip array, which included 65,163 single nucleotide polymorphisms (SNPs), with an average inter-marker distance of 40 kb. Pig individuals with an average call rate below 90% were removed from this study. SNPs were removed if any of the following conditions was met: (1) with call rate <90%; (2) with minor allele frequency (MAF) ≤0.05; (3) did not conform to Hardy-Weinberg equilibrium (HWE) (*i.e.*, multiple tests adjusted p<10^−5^) in any breed; and (4) not included in the latest reference assembly of the porcine genome Sscrofa 10.2. After filtering, 44,433 autosomal SNPs were remained for further analysis.

### Relationship test

To ensure independence among the collected pigs, cryptic relatedness among individuals within each breed were identified using pair wise Identity-By-Descent (IBD) metric (referred to as PI-HAT in PLINK [14]). One individual from a pair of pigs were removed from the following analyses, if their PI-HAT value was over 0.5.

### Principal component analysis

To avoid influence of linkage disequilibrium on principal component analysis, only 21,188 SNPs with pair-wise r^2^<0.2 were actually used in this analysis. The principal component analysis was performed using R package *SNPRelate* [15] to test whether population stratification was present within the sampled animals, and the individuals outside of their expected breed clusters were excluded from further analysis.

### Detection of genomic regions under selection

To identify the genomic selective signatures related to altitude adaptation for TBP and DHP, we performed two separated analyses for the two breeds, both compared with the low-land breed, WZSP. The unbiased estimate of *F_ST_* as described by Weir and Cockerham [16] was calculated using Genepop 4.2 software [17] to identify genome wide spots or patterns for positive selection. To assess statistical significance, we first carried out permutation test (100 permutations). For each replicate the individuals were randomly assigned to one of two breed groups. The null hypothesis is that all rearrangements of the alleles among the two populations are equally probable. The permutation p-value (*P_P_*), as computed previously [18], is the probability that the value of *F_ST_* in the null-distribution exceeds the observed value of *F_ST_*. And then, we obtained the empirical *p*-value (*P*_E_) based on the distribution of nongenic SNPs on which selection is deemed to be weak, since selection preferentially targets gene regions. The empirical *p*-value for *F_ST_* was calculated as follows:
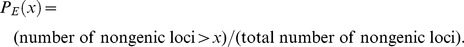



Here, the nongenic SNPs were defined as those located in genomic regions that are at least 50 kb away from the start or stop position of all known genes in pig genome. The 50 kb buffer was used to avoid the linkage with some genes or promoter regions. To further control false positive due to multiple tests for massive genomic SNPs, a Bonferroni corrected significance level (roughly equal to 0.01/number of SNPs analyzed) was used to assess statistical significance. SNPs with *P_P_*<Bonferroni corrected significance level, and also *P_E_*<0.01 were considered as the promising selection signatures. Furthermore, to account for stochasticity in single SNPs, we then clustered all significant SNPs within 500 kb of each other into single highly differentiated regions.

### Bioinformatics analysis

Genes located in the genomic regions significantly differentiated between pig breeds were acquired by the use of the data mining tool Biomart, with the latest reference assembly of the porcine genome Sscrofa10.2 as the reference. Then, human homologous genes were retrieved by using the ‘Homolog filters’ option. Genes without functional annotations or human orthologs were removed. The final homolog list was analyzed by using the online DAVID database tool. In this functional analysis, statistical significance was assessed by using *P* value of a modified Fisher’s exact test and Benjamini correction for multiple tests. Furthermore, a comprehensive literature review was conducted to verify whether these genes have some relevance with adaptive phenotypes in pigs or other mammals.

## Results

### Data preprocessing

A total of 61,565 SNPs were genotyped by using Porcine 60 K Beadchip, of which 9,093 markers were not annotated to genomic porcine genome Sscrofa10.2. For this analysis, data for chromosome X were not included. A total of 44,433 autosomal markers passed the quality control for SNP call rate, consistency with HWE and MAF. The average physical distance between two adjacent SNPs were 54.50 kb, ranging from 46.12 kb on chromosome 12 to 64.59 kb on chromosome 8 (**Table S1**). One DHP individual with >10% missing data was excluded from further analysis. Two pairs of individuals in both TBP and WZSP were found related (with a PI-HAT value of over 0.5), and therefore only one individual from these pairs was randomly selected, yielding the final sample consisting of 91 animals.

### Principle component analysis

**Figure 1:**
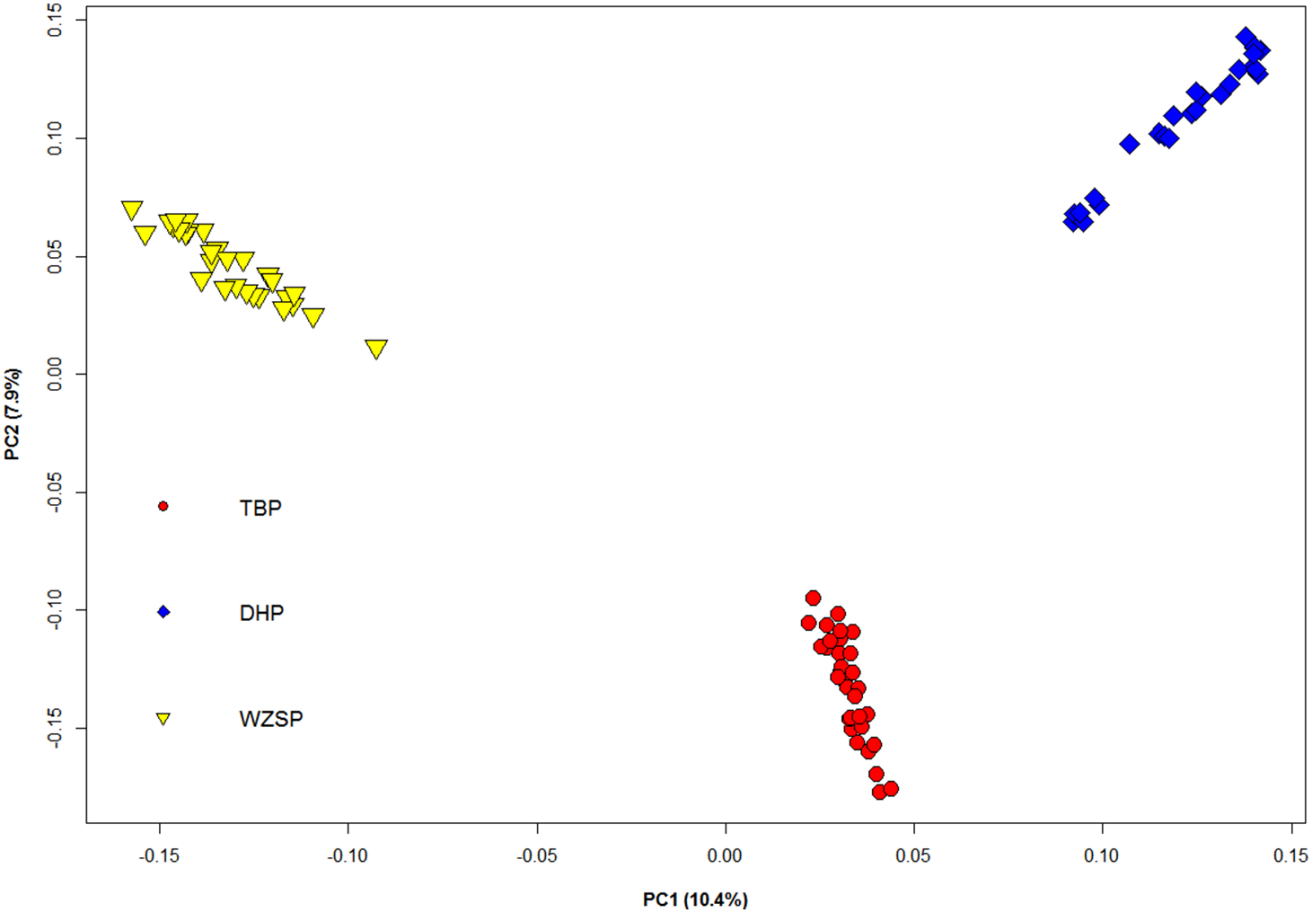
Principle component analysis of TBP, DHP and WZSP individuals. TBP, Tibetan pig; DHP, Dahe pig; WZSP, Wuzhishan pig.

The principle component analysis using a subset of 21,188 SNPs with pair-wise r^2^<0.2 showed that the first two principal components (PC1 and PC2), which explained 10.4% and 7.9% of the variance, respectively, clearly separated the three pig breeds according to their geographic origins. As shown in **Figure 1**, PC1 provided a clear-cut division between two plateau pigs (TBP and DHP) and the low-land pigs (WZSP), while PC2 provided a good separation between TBP and DHP. As none was located outside of its expected breed clusters, all the 91 individuals were kept for further analyses.

### Genome-wide detection of significant SNPs

**Figure 2:**
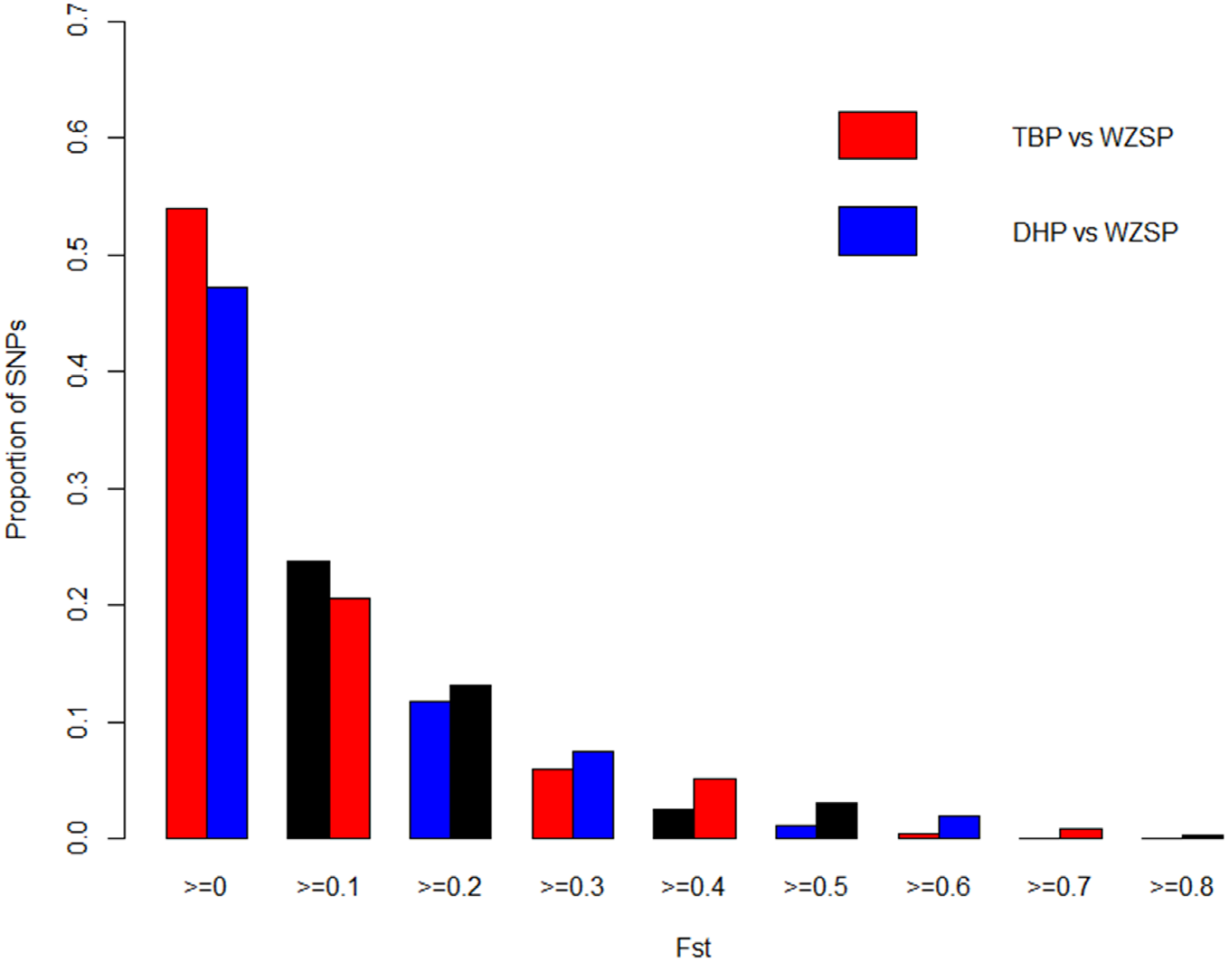
Distributions of *F_ST_* values in different breed comparisons. TBP, Tibetan pig; DHP, Dahe pig; WZSP, Wuzhishan pig.

We calculated *F_ST_* for 37,893 and 32,729 SNPs in TBP vs WZSP and DHP vs WZSP breed comparisons, respectively. The genome-wide distribution of *F_ST_* values for the two analyses was shown in **Figure 2**. In general, higher proportion of large *F_ST_* values (*F_ST_* bins≥0.2) was observed in the breed pair DHP vs WZSP compared to TBP vs WZSP, reflecting that DHP is relatively more diverged from WZSP. The permutation test showed that the observed *F_ST_* distributions for both breed comparisons markedly deviated the permutated ones. The maximum *F_ST_* produced by the permutation was 0.362 for TBP vs WZSP and 0.450 for DHP vs WZSP, respectively, corresponding to the cutoffs for Bonferroni corrected significant levels (*P* = 2.64E-07 and 3.06E-07, respectively) at α = 0.01. Jointly considering the empirical p-value (*P_E_*≤0.01), a total of 395 (observed *F_ST_*≥0.557) and 365 (observed *F_ST_*≥0.716) significant SNPs were obtained in the two breeds comparisons (see **Table S2** and **Table S3** for full list of these SNPs). And then, all significant SNPs within 500 kb of each other were merged into single highly differentiated regions for detection of selective sweeps or genomic regions containing biologically interesting genes, as described below.

### Adaptive signals of selective sweeps for TBP

**Figure 3:**
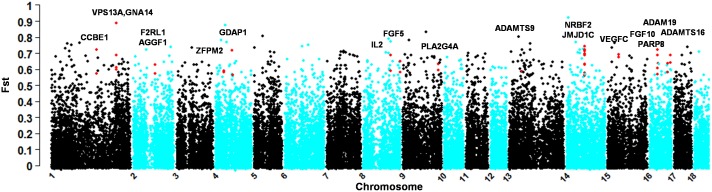
Genomic *F_ST_* distribution of selective signatures identified in comparison of TBP and WZSP. Red dots represent significant sites (*F_ST_*≥0.362, and *P_E_*≤0.01, data not shown) within selected regions containing adaptive genes.

For TBP, a total of 53 highly differentiated regions (selective sweeps) containing two or more SNPs were derived (**Table 1**). Totally, these regions contain 100 genes, of which 18 genes were found having some known biological functions associated with high altitude adaptation, including *CCBE1*, *VPS13A*, *GNA14*, *F2RL1*, *AGGF1*, *ZFPM2*, *GDAP1*, *IL2*, *FGF5*, *PLA2G4A*, *ADAMTS9*, *NRBF2*, *JMJD1C*, *VEGFC*, *FGF10*, *PARP8*, *ADAM19* and *ADAMTS16* (see **Figure 3** for their genomic distributions).

**Table 1:** Genomic regions under selection identified in comparison of TBP and WZSP.

Chr	Region (Mb)	Max *F_ST_*	Min *P_P_*	Min *P_E_*	Genes within selected regions
1	35.37–35.77	0.565	p<2.64E-07	9.34E-03	*ENSSSCG00000028817, ENPP3, ENSSSCG00000004197, ENSSSCG00000023740, ARG1, ENSSSCG00000004196, 7SK, ENSSSCG00000029065, ARG1*
1	42.80–42.83	0.604	p<2.64E-07	5.37E-03	
1	70.70–70.82	0.714	p<2.64E-07	8.06E-04	
1	77.86–78.30	0.750	p<2.64E-07	3.36E-04	
1	178.80–179.00	0.721	p<2.64E-07	6.05E-04	*PMAIP1, * ***CCBE1***
1	257.07–257.20	0.887	p<2.64E-07	0.00E+00	***VPS13A*** *, * ***GNA14***
1	267.89–268.36	0.632	p<2.64E-07	3.36E-03	*C9orf156, HEMGN, ANP32B, NANS, TRIM14, CORO2A, TBC1D2, ENSSSCG00000027558*
1	278.53–278.64	0.632	p<2.64E-07	3.36E-03	
2	87.34–87.73	0.628	p<2.64E-07	3.63E-03	***F2RL1*** *, S100Z, CRHBP, * ***AGGF1*** *, ZBED3, SNORA47, ENSSSCG00000014096*
2	137.74–138.40	0.583	p<2.64E-07	7.59E-03	*ENSSSCG00000021428, ENSSSCG00000014261, CHSY3*
3	6.555–6.558	0.587	p<2.64E-07	7.12E-03	
4	33.81–34.26	0.590	p<2.64E-07	6.85E-03	***ZFPM2***
4	65.95–66.09	0.566	p<2.64E-07	9.07E-03	
4	66.95–67.20	0.717	p<2.64E-07	6.72E-04	***GDAP1***
4	141.58–142.01	0.591	p<2.64E-07	6.79E-03	*ENSSSCG00000022032, ENSSSCG00000030125, SH3GLB1*
5	55.36–55.78	0.647	p<2.64E-07	2.69E-03	*SLCO1A2, ENSSSCG00000021998, SLCO1C1*
6	109.79–109.86	0.625	p<2.64E-07	3.83E-03	*ASXL3*
7	56.15–56.23	0.708	p<2.64E-07	1.01E-03	
7	65.80–65.98	0.715	p<2.64E-07	7.39E-04	
7	125.9429–125.9430	0.645	p<2.64E-07	2.82E-03	
8	27.12–27.49	0.569	p<2.64E-07	8.67E-03	
8	57.74–57.75	0.589	p<2.64E-07	6.92E-03	
8	91.21–91.35	0.703	p<2.64E-07	1.21E-03	*ENSSSCG00000009052*
8	102.21–103.34	0.791	p<2.64E-07	1.34E-04	*ENSSSCG00000027713, C4orf33, ENSSSCG00000009070, JADE1, ENSSSCG00000029943, ENSSSCG00000027234*
8	105.18–105.46	0.622	p<2.64E-07	4.37E-03	
8	108.70–109.10	0.686	p<2.64E-07	1.48E-03	***IL2*** *, ADAD1, KIAA1109*
8	146.65–146.73	0.650	p<2.64E-07	2.62E-03	***FGF5***
9	22.97–23.41	0.780	p<2.64E-07	2.69E-04	*FZD4, ENSSSCG00000024272, ENSSSCG00000014922*
9	93.10–93.21	0.833	p<2.64E-07	0.00E+00	
9	122.78–123.11	0.671	p<2.64E-07	2.02E-03	
9	140.59–140.74	0.631	p<2.64E-07	3.36E-03	***PLA2G4A***
11	2.72–2.87	0.618	p<2.64E-07	4.57E-03	*ENSSSCG00000027577*
11	6.14–6.16	0.683	p<2.64E-07	1.68E-03	*MTUS2*
11	8.14–8.56	0.702	p<2.64E-07	1.34E-03	*ENSSSCG00000009336, FRY, ENSSSCG00000009337*
11	68.77–69.16	0.671	p<2.64E-07	2.08E-03	
13	46.62–46.65	0.686	p<2.64E-07	1.55E-03	
13	50.67–50.69	0.721	p<2.64E-07	6.72E-04	***ADAMTS9***
13	60.17–60.18	0.702	p<2.64E-07	1.41E-03	
13	60.98–61.21	0.619	p<2.64E-07	4.50E-03	
13	82.47–82.50	0.759	p<2.64E-07	3.36E-04	*SRPRB*
14	16.30–16.54	0.626	p<2.64E-07	3.83E-03	*DEFB134, ENSSSCG00000026659*
14	44.86–44.98	0.613	p<2.64E-07	4.77E-03	*CMLKR1*
14	53.16–53.24	0.721	p<2.64E-07	6.05E-04	*ENSSSCG00000010062, ENSSSCG00000010063*
14	71.80–72.93	0.742	p<2.64E-07	4.03E-04	***NRBF2*** *, * ***JMJD1C*** *, ssc-mir-1296, ENSSSCG00000023310, REEP3, ENSSSCG00000028393*
15	44.40–44.86	0.691	p<2.64E-07	1.48E-03	*SPATA4, ASB5, SPCS3, * ***VEGFC***
15	87.54–87.64	0.604	p<2.64E-07	5.44E-03	*HAT1, METAP1D, DLX1*
15	95.12–95.92	0.671	p<2.64E-07	2.08E-03	*CWC22*
15	150.24–150.37	0.626	p<2.64E-07	3.83E-03	*ENSSSCG00000016317*
16	30.26–30.96	0.723	p<2.64E-07	6.05E-04	***FGF10*** *, HCN1*
16	31.61–31.98	0.637	p<2.64E-07	3.29E-03	*SNORD28, EMB, * ***PARP8***
16	68.45–68.47	0.623	p<2.64E-07	4.30E-03	*PTTG1*
16	71.35–71.75	0.638	p<2.64E-07	3.29E-03	*CLINT1, U6, LSM11, THG1L, SOX30, U6, * ***ADAM19*** *, ENSSSCG00000028459, NIPAL4, CYFIP2*
16	82.68–82.84	0.688	p<2.64E-07	1.48E-03	***ADAMTS16*** *, ENSSSCG00000026531*

Notes: *P_P_*, permutation p-value, and for comparison of TBP and WZSP, the Bonferroni corrected significant level (at α = 0.01) for *P_P_* = 0.01/37, 893 (# of SNPs analyzed) = 2.64E-07; *P_E_*, empirical p-value; Adaptive genes that have plausible biological functions contributing local adaptation are in bold.

Hereafter, we focus on reviewing the 18 promising genes, of which three genes, *CCBE1*, *VPS13A* and *GNA14*, are situated on chromosome 1, and all have potential relevance to the physiological functions for altitude adaptation. *CCBE1* contributes to the lymphatic vascular development [19], while *VPS13A* was known to be associated with cell membrane deformation of circulating erythrocytes [20]. Another gene, *GNA14*, was previously shown to be induced by hypoxia and plays an important role in placental and fetal vascular endothelial functions under chronic hypoxia [21] (http://www.erp.wisc.edu/symposium/2012_abstracts.pdf), although the precise mechanisms of its response to hypoxia was not completely clear. The selective sweep on chromosome 2 (genomic location: 87.34–87.73 Mb) harbors two interesting functional genes, *F2RL1* and *AGGF1*. Genetic variants in *F2RL1* was found to be associated with blood pressure [22], and AGGF1 is an angiogenic factor and essential for embryonic and pathological angiogenesis [23]. Two promising selection signatures on chromosome 4 are *ZFPM2*, which plays a critical role in heart development and coronary vascular development [24], and *GDAP1*, which is associated with mitochondrial fission [25]. Two selective sweeps on chromosome 8 harbor two interesting genes, of which *IL2* (genomic location: 108.70–109.10 Mb) is an important molecule for maintaining natural immunologic self-tolerance [26], and *FGF5* (genomic location: 146.65–146.73 Mb) plays a role in increasing blood flow and contractile function in an ischemic region of the heart [27]. An interesting selective gene was also found on chromosomes 9 and 13. *PLA2G4A*, located on chromosome 9 (genomic location: 140.59–140.74 Mb), contributes to the pulmonary vasoconstrictor response to hypoxia [28], and *ADAMTS9* on chromosome 13 (genomic location: 50.67–50.69 Mb), an essential element for normal cardiovascular development and adult homeostasis [29], was also identified to be highly selective in a previous study of TBP [9]. Four selective regions on chromosome 14 harbor two interesting genes, *NRBF2* and *JMJD1C*. *NRBF2* was deemed to be an important molecule for exercise-mediated mitochondrial expansion and altered fuel selection, which enhances aerobic ATP-generating capacity in skeletal muscle [30]. *JMJD1C*, encoding a probable histone demethylase, was found to be involved in hematopoiesis in humans and mouse [31]. Interestingly, *VEGFC* on chromosome 15 and *FGF10* on chromosome 16 were also identified in a previous study of TBP [9]. *VEGFC*, a vascular endothelial growth factor [32], is particular interesting as it is involved in hypoxia-inducible factor (HIF) pathway that is initiated in response to hypoxic environmental conditions by regulating oxygen homeostasis in humans and other mammals [33]. And *FGF10* plays a crucial role in lung branching morphogenesis during early embryonic lung development. Finally, three additional interesting genes were found on chromosome 16, of which *PARP8* is related to DNA repair [34], *ADAM19* plays essential roles in cardiovascular morphogenesis and heart development [35], [36] and *ADAMTS16* is associated with blood pressure [37].

To further elucidate the functional involvements of the 100 selective genes, we performed a GO-based enrichment analysis using the DAVID database tools. A total of 12 functional categories achieved the nominal significance at α = 0.05 (as shown in **Table 2**), based Fisher’s exact test. However, they did not reach the highly conservative Benjamini multiple-tests corrected level. These categories were mainly involved in cardiovascular system, including “blood vessel morphogenesis” (*P* = 0.014, GO: 0048514), “blood vessel development” (*P* = 0.023, GO: 0001568), “vasculature development” (*P* = 0.025, GO: 0001944) and “angiogenesis” (*P* = 0.028, GO: 0001525), reflecting the adaptive response of developed blood vessels to increase the efficiency of oxygen utilization in TBP. In addition, categories involved in “positive regulation of response to external stimulus” (*P* = 0.033, GO: 0032103) and “regulation of response to external stimulus” (*P* = 0.033, GO: 0032101) were significantly overrepresented, which may reflect the adaptive response of TBP to the external stresses of low oxygen concentration and strong ultraviolet radiation at the high-altitude plateau. Significant GO terms associated with breeding traits such as “gamete generation” (*P* = 0.008, GO: 0007276) may reflect the effort of TBP to maintain an adequate fitness in the harsh high altitude environment by maximize its reproduction success.

**Table 2:** Functional enrichment analysis of genes within the selected regions identified in comparison of TBP and WZSP.

GO term	GO category	Gene number	p-value	Benjamini
GO:0007276	gamete generation	7	0.008	0.998
GO:0048514	blood vessel morphogenesis	5	0.014	0.996
GO:0019953	sexual reproduction	7	0.016	0.984
GO:0048609	reproductive process in a multicellular organism	7	0.020	0.983
GO:0032504	multicellular organism reproduction	7	0.020	0.983
GO:0001568	blood vessel development	5	0.023	0.975
GO:0001944	vasculature development	5	0.025	0.965
GO:0001525	angiogenesis	4	0.028	0.959
GO:0032103	positive regulation of response to external stimulus	3	0.033	0.962
GO:0032101	regulation of response to external stimulus	4	0.033	0.950
GO:0048232	male gamete generation	5	0.048	0.979
GO:0007283	spermatogenesis	5	0.048	0.979

Notes: GO term, subcategory of biological process.

### Adaptive signals of selective sweeps for DHP

**Figure 4:**
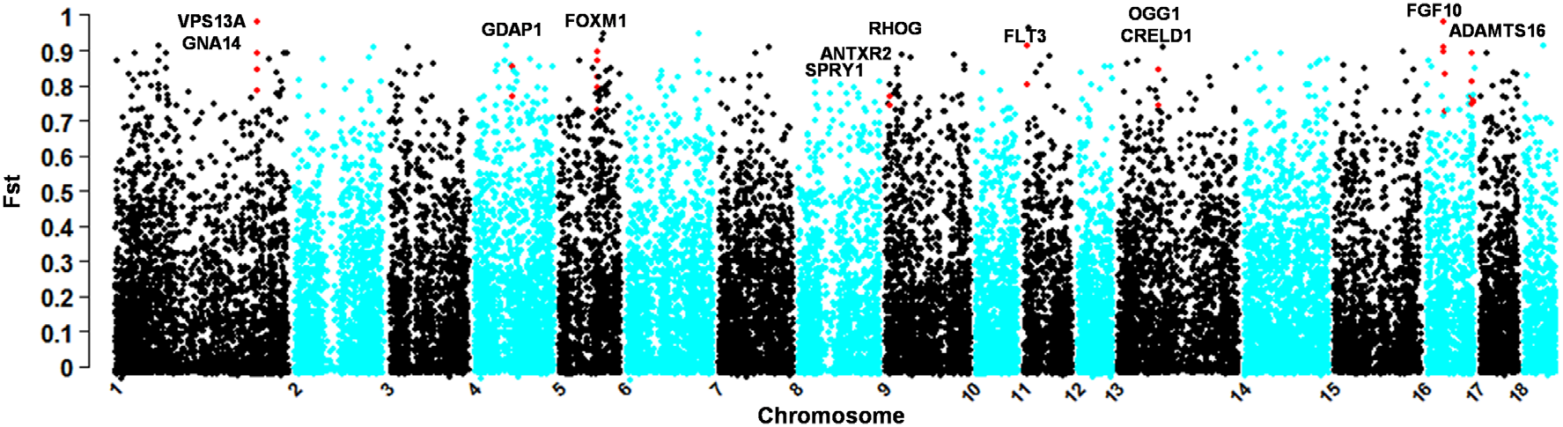
Genomic distribution of signatures of selection identified in comparison of DHP and WZSP. Red dots represent significant sites (*F_ST_*≥0.450, and *P_E_*≤0.01, data not shown) within selected regions containing adaptive genes.

To determine whether the moderate altitude environments could also trigger the genetic adaptation to mild hypoxia, we further analyzed the genomic *Fst* profile of selection for DHP, a breed living at an altitude of about 2000 meters, compared with the low-land breed WZSP. The 365 significant SNPs mapped onto 42 genomic regions defined as the selective sweeps (**Table 3**). These regions encompass 137 genes, of which 12 genes were found having known biological functions related to altitude adaptation and were illustrated in **Figure 4**. Interestingly, six genes were overlapped with the list of genes identified in TBP as mentioned above, including *VPS13A*, *GNA14*, *GDAP1*, *PARP8*, *FGF10* and *ADAMTS16*.

**Table 3:** Genomic regions under selection identified in comparison of DHP and WZSP.

Chr	Region (Mb)	Max *F_ST_*	Min *P_P_*	Min *P_E_*	Genes within selected regions
1	33.72–34.18	0.891	p<3.06E-07	7.73E-04	*ENSSSCG00000023999*
1	41.84–41.99	0.795	p<3.06E-07	3.94E-03	*RNF217*
1	65.31–65.32	0.798	p<3.06E-07	3.56E-03	
1	77.80–78.50	0.912	p<3.06E-07	2.32E-04	
1	91.68–92.00	0.891	p<3.06E-07	7.73E-04	*FRK, NT5DC1, COL10A1*
1	95.87–96.06	0.759	p<3.06E-07	5.80E-03	
1	256.78–257.14	0.982	p<3.06E-07	0.00E+00	***VPS13A*** *, * ***GNA14***
1	278.35–278.82	0.875	p<3.06E-07	1.00E-03	*ENSSSCG00000023284, ZNF462*
1	297.24–297.27	0.776	p<3.06E-07	5.02E-03	*ENSSSCG00000005575, SNORD90*
2	137.19–137.24	0.763	p<3.06E-07	5.64E-03	*ISOC1*
3	12.09–12.23	0.767	p<3.06E-07	5.49E-03	
3	55.80–56.01	0.853	p<3.06E-07	1.62E-03	*RPL31, TBC1D8, NPAS2*
4	17.65–17.65	0.768	p<3.06E-07	5.33E-03	
4	57.71–57.84	0.912	p<3.06E-07	2.32E-04	*ENSSSCG00000027870, U6*
4	65.95–66.12	0.858	p<3.06E-07	1.31E-03	
4	67.06–67.20	0.851	p<3.06E-07	1.62E-03	***GDAP1***
4	107.28–107.52	0.749	p<3.06E-07	6.57E-03	*PIP5K1A, VPS72, TMOD4, SCNM1, LYSMD1, TNFAIP8L2, SEMA6C, 7SK, GABPB2, MLLT11, CDC42SE1, C1orf56, BNIPL, PRUNE, FAM63A, ANXA9, CERS2, SETDB1*
4	126.98–127.07	0.789	p<3.06E-07	4.56E-03	
5	68.78–69.69	0.896	p<3.06E-07	6.18E-04	*PARP11, PRMT8, EFCAB4B, ENSSSCG00000000734, TSPAN9, TEAD4, ENSSSCG00000030266, TULP3, RHNO1, * ***FOXM1*** *, ENSSSCG00000000740, NRIP2, ITFG2, FKBP4, ENSSSCG00000000744, DDX11, WASH4P, * ***RTEL1***
5	71.82–71.89	0.822	p<3.06E-07	2.86E-03	
5	75.58–76.06	0.930	p<3.06E-07	1.55E-04	*5S_rRNA, PDZRN4*
5	84.38–84.48	0.840	p<3.06E-07	2.01E-03	*ENSSSCG00000000854*
5	86.56–86.77	0.785	p<3.06E-07	4.71E-03	*MYBPC1, SPIC*
6	7.64–8.02	0.765	p<3.06E-07	5.64E-03	*CDYL2*
8	57.78–58.00	0.778	p<3.06E-07	4.87E-03	*ARL9, SRP72, PAICS, PPAT, AASDH, KIAA1211*
8	107.95–108.12	0.776	p<3.06E-07	5.02E-03	*snoU13, SPRY1*
8	146.73–146.94	0.811	p<3.06E-07	3.17E-03	*ENSSSCG00000024283, ENSSSCG00000020770, PRDM8, ANTXR2*
9	7.07–7.12	0.767	p<3.06E-07	5.49E-03	***RHOG*** *, NUP98*
9	21.27–21.68	0.832	p<3.06E-07	2.40E-03	
9	22.73–22.99	0.850	p<3.06E-07	2.01E-03	*PRSS23*
9	106.49–106.56	0.768	p<3.06E-07	5.41E-03	
9	138.75–138.76	0.771	p<3.06E-07	5.18E-03	*TRMT1L*
9	143.77–144.24	0.858	p<3.06E-07	1.31E-03	*ENSSSCG00000026587, U6, NENF, TMEM206, PPP2R5A, U6*
11	4.91–5.22	0.912	p<3.06E-07	2.32E-04	*ENSSSCG00000009312, * ***FLT3*** *, ENSSSCG00000023944, ENSSSCG00000009315, URAD, CDX-2, PAN3*
13	73.02–73.29	0.844	p<3.06E-07	2.01E-03	*MTMR14, TADA3, CAMK1, * ***OGG1*** *, BRPF1, CPNE9, ARPC4, ENSSSCG00000011553, RPUSD3, CIDE-C, JAGN1, IL17RE, IL17RC, * ***CRELD1*** *, PRRT3*
13	210.65–210.84	0.734	p<3.06E-07	7.57E-03	*HLCS, RIPPLY3, PIGP, TTC3, ENSSSCG00000020975*
14	28.19–28.55	0.851	p<3.06E-07	1.86E-03	*ENSSSCG00000009753*
14	118.40–118.81	0.803	p<3.06E-07	3.32E-03	*UBTD1, ANKRD2, HOGA1, MORN4, PI4K2A, ENSSSCG00000020874, AVPI1, MARVELD1, ZFYVE27, SFRP5, ENSSSCG00000010530*
16	30.26–30.52	0.982	p<3.06E-07	0.00E+00	***FGF10***
16	31.98–32.18	0.830	p<3.06E-07	2.47E-03	***PARP8***
16	82.43–83.81	0.891	p<3.06E-07	7.73E-04	*ICE1, ENSSSCG00000017107, * ***ADAMTS16*** *, ENSSSCG00000026531, U6, ENSSSCG00000017110*
17	41.27–41.59	0.813	p<3.06E-07	3.09E-03	*DNMT3B, MAPRE1, ENSSSCG00000029351, SPAG4L, BPIFB2, BPIFB6, BPIFB4, BPIFB3*

Notes: *P_P_*, permutation p-value, and for comparison of DHP and WZSP, the Bonferroni corrected significant level (at α = 0.01) for *P_P_* = 0.01/32, 729 (# of SNPs analyzed) = 3.06E-07; *P_E_*, empirical p-value; Adaptive genes that have plausible biological functions contributing local adaptation are in bold.

Six additional genes were specific to DHP, including *FOXM1*, *RTEL1*, *RHOG*, *FLT3*, *OGG1* and *CRELD1*. *FOXM1* and *RTEL1*, both located on a specific genomic region of chromosome 5 (68.78–69.69 Mb), were reported to play an essential role in repairing oxidative DNA damage [38], [39]. *RHOG*, located on chromosome 9 (genomic location: 7.07–7.12 Mb), is an important regulator for reactive oxygen species (ROS) production and erythropoiesis [40]. *FLT3*, located on chromosome 11 (4.91–5.22 Mb) and *OGG1*, located on chromosome 13 (73.02–73.29 Mb), are also associated with repair of oxidative DNA damage [41]. Furthermore, *FLT3* has an additional function as a regulator for ROS-producing [42] and hematopoiesis [43]. Another gene also within the chromosome 13 sweep (73.02–73.29 Mb), *CRELD1*, was shown to be associated with cardiac atrioventricular septal defects [44].

Similar to the above analysis for TBP, further GO-based analysis of these highly divergent genes did not reveal any functional categories reaching the Beniamini corrected level for statistical significance. However, 14 GO categories related to nucleus and chromosome organization were of nominally significance with a modified Fisher’s *p*-value<0.05 (**Table 4**). These functional terms were known to be associated with DNA repair and genome maintenance [45], and these findings may reflect the functional changes of DHP in order to adapt well to the harsh environments such as low ambient oxygen, increased risk of oxidative stress, and strong ultraviolet radiation.

**Table 4:** Functional enrichment analysis of genes within selected regions identified in comparison of DHP and WZSP.

GO term	GO category	Gene number	p-value	Benjamini
GO:0006144	purine base metabolic process	3	0.002	0.780
GO:0016568	chromatin modification	7	0.003	0.630
GO:0006325	chromatin organization	8	0.004	0.536
GO:0051276	chromosome organization	9	0.004	0.453
GO:0009112	nucleobase metabolic process	3	0.006	0.559
GO:0008284	positive regulation of cell proliferation	7	0.021	0.899
GO:0042127	regulation of cell proliferation	10	0.021	0.861
GO:0016569	covalent chromatin modification	4	0.029	0.900
GO:0001708	cell fate specification	3	0.029	0.879
GO:0046040	IMP metabolic process	2	0.031	0.867
GO:0006189	‘de novo’ IMP biosynthetic process	2	0.031	0.867
GO:0006188	IMP biosynthetic process	2	0.031	0.867
GO:0031503	protein complex localization	2	0.036	0.882
GO:0009113	purine base biosynthetic process	2	0.046	0.919

Notes: GO term, subcategory of biological process.

### The selective sweeps and genes shared by TBP and DHP

As mentioned above, six genes (*VPS13A*, *GNA14*, *GDAP1*, *PARP8*, *FGF10* and *ADAMTS16*) from completely or partly overlapped genomic regions for selective sweeps were shared by TBP and DHP (**Table 5**). The first overlapped region is located on chromosome 1, covering a 0.07 Mb segment (from 257.07 to 257.14 Mb). There were four significant SNP signals in TBP, and two in DHP, of which two SNP loci (INRA0006383 and H3GA0003828) were duplicated in the two breeds. Based on the biological functions of *VPS13A* and *GNA14* located in this genomic region, both breeds might follow a similar route to adapt under chronic hypoxia. The second overlapped region is situated on chromosome 4, and 0.15 Mb long (from 67.05 to 67.20 Mb). One identical locus (ALGA0025367) was found in both breeds, although DHP had larger *Fst* values. This region accommodates *GDAP1* (location: 66.95–67.20), a gene encoding ganglioside-induced differentiation-associated protein 1. All the remaining three shared sweep regions are situated on chromosome 16. For the sweep ranging from 30.26 to 30.52 Mb, two of three selective SNP signals (DRGA0016021 and DRGA0016027) were repeatly identified in the two breeds. As the contained gene *FGF10* reveals, this shared region may implicate of a similar adaptive physiological and development changes in the lungs of the two pig breeds. Only a single selective SNP (ALGA0090039) was identified in the two breeds on the fourth sweep and its associated gene was *PARP8*, which is related to DNA repair. However, for the sweep region ranging from 82.68 to 82.84 Mb, two different SNP signals (M1GA0021378 and ALGA0092291) were observed in the two breeds. This region may be associated with pigs’ adaptive blood pressure changes to high altitudes, inferred from the gene (*ADAMTS16*) located in this region. Overall, these shared sweep regions provide an avenue for us to further unveil the common molecular mechanisms for the local adaptation to high altitude environments.

**Table 5:** Summary of shared selective sweeps.

Overlapping regions (Mb)	SNPs in regions	Position (bp)	*F_ST_* values		Promising genes in regions
			TBP vs WZSP	DHP vs WZSP	
Chr1: 257.07–257.14	INRA0006383	257072211	0.887	0.982	*VPS13A*, *GNA14*
	H3GA0003822	257096974	0.612	0.611	
	ASGA0006012	257136966	0.530	0.844	
	H3GA0003828	257175271	0.685	0.709	
	ASGA0006016	257198514	0.598	0.693	
Chr4: 67.05–67.20	INRA0014374	67057104	0.556	0.851	*GDAP1*
	ALGA0025367	67199718	0.563	0.770	
Chr16: 30.26–30.52	DRGA0016021	30264193	0.723	0.896	*FGF10*
	MARC0105115	30377127	0.505	0.982	
	DRGA0016027	30515922	0.568	0.909	
Chr16: 31.98–31.98	ALGA0090039	31983325	0.637	0.830	*PARP8*
Chr16: 82.68–82.84	M1GA0021378	82675301	0.641	0.220	*ADAMTS1*6
	ALGA0092291	82835496	0.688	0.042	

## Discussion

In the present study, we identified genomic selective sweeps in TBP and DHP using a dense genome wide panel of SNPs, with a major focus on the detection of signals relevant for altitude phenotypes. Generally, an *F_ST_* above 0.25 indicates salient differentiation between two populations [46]. However, the *F_ST_* values in the significant SNP lists for both breed comparisons all were above 0.5, indicating very high divergence between these pig breeds. Similar degree of divergence were also observed between Northern and Southern Chinese indigenous pig breeds in a recent report by Yang et al. [47]. In our own view, these results are not very surprising, as many Chinese native pig breeds have lived in highly isolated niches for long time. Nevertheless, we could not deny other evolutionary forces than the natural selection and genetic drift occurred in these ecological niches. Consequently, differences in the adaptive traits associated with these selective sweeps may also be due to differences in production, reproduction, and survivability which may have been emphasized during breed formation and/or selected for over time.

Another notable finding is that DHP appears to be more diverged from the low-land breed WZSP than TBP, based on their corresponding *F_ST_* distributions (**Figure 2**) and the longer list of significant SNPs for DHP. This pattern is not what we expected since TBP is deemed to suffer from higher level of environmental pressure. This unexpected result might be due to several reasons. First, DHP is distributed in a very restricted region in Yunnan province and its population size have decreased dramatically in history, which may generate a strong bottle-neck effect [48]. Currently, only a limited number of DHP are raised in several conservation farms. The smaller effective population size of DHP, compared with that of TBP, may lead to more genetic divergence. Second, some artificial breeding practice occurred in these farms for conserving this breed in recent years may also accelerate its genetic divergence from the low-land breed WZSP.

Despite TBP has evolved excellent adaptive capabilities to the high-altitude Qinghai-Tibetan plateau, its genetic basis remains not very clear. Although two recent studies have proposed a set of candidate genes, there is no consensus regarding their importance for the local adaptation. Ai et al. [8] have highlighted three candidate genes (*ADAMTS12*, *SIM1* and *NOS1*) that are likely important for adaptation to high altitude, which, unfortunately, were not detected in both our study and another previous study [9]. The sampling variations and the different methods to capture the genomic traces may be able to explain these incongruities. Nevertheless, three genes, *VEGFC*, *FGF10* and *ADAMTS9*, previously identified by Li et al [9], were replicated by the present study, all having sounding biological functions. Furthermore, *VEGFC* was also included in a set of positively-selected genes for andeans [49], [50], suggesting that this gene is a key element for multiple species.

Of the 18 selective genes identified in TBP, 12 (*CCBE1, F2RL1, AGGF1, ZFPM2, IL2, FGF5, PLA2G4A, ADAMTS9, NRBF2, JMJD1C*, *VEGFC* and *ADAM19*) appear to be specific to TBP, by compared with the results for DHP. Those TBP-specific genes (*i.e.*, *CCBE1*, *ZFPM2*, *AGGF1*, *PLA2G4A*, *ADAMTS9*, *VEGFC*, and *ADAM19*) have potential roles in many biological processes or physiological processes, in particular, related to cardiovascular conditioning. This observation is consistent with the fact that TBP has larger lungs and hearts [9], which allow for more efficient oxygen utilization. In addition, we can assume that nature selection acting on genes involved in hematological parameters (*i.e.*, *FGF5*, *JMJD1C* and *F2RL1*), energy metabolism (*i.e.*, *NRBF2*) and immune function (*i.e.*, *IL2*) may have also contributed to TBP’s local adaptation.

DHP dwells at moderate altitudes of approximately 2000 m in Yunnan province. At this altitude, the standard barometric pressure reduces nearly 20% when compared to the sea level [12]. Previous studies have proved that residence at moderate altitude may also trigger some physiological changes due to the selective pressure of hypoxia. For example, birth weight declined significantly with the elevation of altitude from 1500 m to 2000 m [51], indicating that moderate-altitude has profound effects on fetal growth. Previous studies [52], [53] also provided evidence of genetic adaptation to moderate-altitude in humans. However, to our knowledge, there is no published literature related to genetic adaptation to moderate-altitude in pigs. In this study, we identified 12 candidate genes (*VPS13A*, *GNA14, GDAP1, FOXM1, RTEL1, RHOG, FLT3, OGG1, CRELD1, FGF10, PAPR8* and *ADAMTS16*), which may be responsible for the local adaptation in DHP. Of these genes, six (*FOXM1*, *RTEL1, RHOG*, *FLT3*, *OGG1* and *CRELD1*) were unique to DHP and all have some relevant functions to the local altitude adaptation. Among them, five genes are related to DNA repair for oxidative damage or regulation of ROS generation (*i.e.*, *FOXM1, RTEL1, FLT3*, *OGG1* and *RHOG*). It is known that both the oxidative stress [54]–[56] and ROS [57] would increase at altitude due to hypoxia, cold, or ultra violet light, which may lead to DNA strand breaks and tissue injury [57]. Therefore, these unique genes may have shaped some specific molecular adaptation mechanisms of DHP to the local environment of moderate altitude.

Finally, to test whether pigs dwelling different levels of altitudes share some common adaptive mechanisms, we further compared genome-wide selection profiles for TBP, living at high altitude, and DHP, living at moderate altitude. Although a previous study of humans living in high- and moderate-altitude successfully identified *EGLN1* as a common genetic factor for different levels of altitudes [52], no similar study of pigs is reported so far. Our genome-wide comparison of selective profiles for TBP and DHP yielded six common genes (*VPS13A*, *GNA14*, *GDAP1*, *PARP8*, *FGF10* and *ADAMTS16*). Further literature reviews suggest that TBP and DHP may have some similar adaptive changes in physiology and development, such as developmental changes in lung and vascular endothelial cells etc. However, to further unveil these common molecular mechanisms requires careful designed experiments.

## Supporting Information

Table S1 **Distribution of SNPs in the autosomal chromosomes.**
(DOCX)

Table S2 **List of significant SNPs in comparison of TBP and WZSP.** ***P_P_*** **, permutation p-value;** ***P_E_*** **, empirical p-value.**
(DOCX)

Table S3 **List of significant SNPs in comparison of DHP and WZSP.** ***P_P_*** **, permutation p-value;** ***P_E_*** **, empirical p-value.**
(DOCX)
