# Supplementary material for: Genomic Scan Reveals Loci under Altitude Adaptation in Tibetan and Dahe Pigs
Source: PLoS One. 2014 Oct 17;9(10):e110520. doi: 10.1371/journal.pone.0110520 (PMC4201535; doi:10.1371/journal.pone.0110520)
Supplement: Table S1 — Distribution of SNPs in the autosomal chromosomes. (DOCX) [file pone.0110520.s001.docx]

**Table S1 Distribution of SNPs in the autosomal chromosome**

| **Chr** | **Sizes(Mb)** | **SNP no.** | **Distance(Kb)** | **SNP no.**  **(after filter)** | **Distance**  **(Kb, after filter)** |
| --- | --- | --- | --- | --- | --- |
| 1 | 315.3 | 6356 | 49.61 | 5329 | 59.17 |
| 2 | 162.6 | 3303 | 49.23 | 2877 | 56.52 |
| 3 | 144.8 | 2746 | 52.73 | 2404 | 60.23 |
| 4 | 143.5 | 3492 | 41.09 | 3046 | 47.11 |
| 5 | 111.5 | 2298 | 48.52 | 2003 | 55.67 |
| 6 | 157.8 | 3141 | 50.24 | 2758 | 57.22 |
| 7 | 134.8 | 3264 | 41.30 | 2874 | 46.90 |
| 8 | 148.5 | 2717 | 54.66 | 2299 | 64.59 |
| 9 | 153.7 | 3137 | 49.00 | 2766 | 55.57 |
| 10 | 79.1 | 1752 | 45.15 | 1559 | 50.74 |
| 11 | 87.7 | 1854 | 47.30 | 1652 | 53.09 |
| 12 | 63.6 | 1531 | 41.54 | 1379 | 46.12 |
| 13 | 218.6 | 3998 | 54.68 | 3445 | 63.45 |
| 14 | 153.9 | 3844 | 40.04 | 3333 | 46.17 |
| 15 | 157.7 | 2836 | 55.61 | 2491 | 63.31 |
| 16 | 86.9 | 1836 | 47.33 | 1590 | 54.65 |
| 17 | 69.7 | 1667 | 41.81 | 1480 | 47.09 |
| 18 | 61.2 | 1312 | 46.65 | 1148 | 53.31 |
| X | 144.3 | 1388 | 103.96 | 0 | 0 |
| Unmapped | 9093 | | | | |
| Sum |  | 61565 | 47.58 | 44433 | 54.5 |
