# Supplementary material for: Genomic Scan Reveals Loci under Altitude Adaptation in Tibetan and Dahe Pigs
Source: PLoS One. 2014 Oct 17;9(10):e110520. doi: 10.1371/journal.pone.0110520 (PMC4201535; doi:10.1371/journal.pone.0110520)
Supplement: Table S2 — List of significant SNPs in comparison of TBP and WZSP. PP , permutation p-value; PE , empirical p-value. (DOCX) [file pone.0110520.s002.docx]

**Table S2 List of significant SNPs in comparison of TBP and WZSP**

| **Chr** | **Name** | **Position** | **F_ST_** | ***P_P_*** | ***P_E_*** |
| --- | --- | --- | --- | --- | --- |
| 14 | ALGA0074699 | 7044169 | 0.922 | p<2.64E-07 | 0.00E+00 |
| 1 | INRA0006383 | 257072211 | 0.887 | p<2.64E-07 | 0.00E+00 |
| 4 | INRA0013758 | 39830135 | 0.874 | p<2.64E-07 | 0.00E+00 |
| 9 | H3GA0027860 | 93098631 | 0.833 | p<2.64E-07 | 0.00E+00 |
| 9 | ALGA0054078 | 93125776 | 0.833 | p<2.64E-07 | 0.00E+00 |
| 5 | ALGA0031521 | 33095734 | 0.807 | p<2.64E-07 | 1.34E-04 |
| 13 | ALGA0117401 | 37220929 | 0.803 | p<2.64E-07 | 1.34E-04 |
| 8 | DRGA0008716 | 102205112 | 0.791 | p<2.64E-07 | 1.34E-04 |
| 4 | MARC0069526 | 22259328 | 0.783 | p<2.64E-07 | 2.02E-04 |
| 9 | ALGA0118446 | 22965566 | 0.780 | p<2.64E-07 | 2.69E-04 |
| 8 | ALGA0113244 | 107954353 | 0.774 | p<2.64E-07 | 2.69E-04 |
| 4 | ALGA0024760 | 42953649 | 0.770 | p<2.64E-07 | 2.69E-04 |
| 14 | MARC0113902 | 36699210 | 0.769 | p<2.64E-07 | 2.69E-04 |
| 15 | ASGA0069017 | 28181899 | 0.765 | p<2.64E-07 | 2.69E-04 |
| 1 | INRA0003491 | 109874960 | 0.765 | p<2.64E-07 | 2.69E-04 |
| 13 | DRGA0012610 | 82496275 | 0.759 | p<2.64E-07 | 3.36E-04 |
| 1 | ASGA0002957 | 61940130 | 0.759 | p<2.64E-07 | 3.36E-04 |
| 6 | ASGA0029105 | 95355941 | 0.751 | p<2.64E-07 | 3.36E-04 |
| 1 | DRGA0001131 | 78300780 | 0.750 | p<2.64E-07 | 3.36E-04 |
| 6 | ALGA0115562 | 68428074 | 0.742 | p<2.64E-07 | 4.03E-04 |
| 14 | ASGA0064211 | 72469714 | 0.742 | p<2.64E-07 | 4.03E-04 |
| 2 | DIAS0004579 | 148345545 | 0.738 | p<2.64E-07 | 5.37E-04 |
| 17 | H3GA0049247 | 53856831 | 0.738 | p<2.64E-07 | 5.37E-04 |
| 3 | MARC0044793 | 59501141 | 0.736 | p<2.64E-07 | 5.37E-04 |
| 15 | ASGA0093353 | 15507527 | 0.735 | p<2.64E-07 | 5.37E-04 |
| 5 | ALGA0029883 | 2052182 | 0.735 | p<2.64E-07 | 6.05E-04 |
| 1 | MARC0051272 | 55354812 | 0.731 | p<2.64E-07 | 6.05E-04 |
| 14 | H3GA0040656 | 66437563 | 0.724 | p<2.64E-07 | 6.05E-04 |
| 16 | DRGA0016021 | 30264193 | 0.723 | p<2.64E-07 | 6.05E-04 |
| 13 | ALGA0070941 | 82469840 | 0.722 | p<2.64E-07 | 6.05E-04 |
| 14 | ASGA0063360 | 53156113 | 0.721 | p<2.64E-07 | 6.05E-04 |
| 1 | MARC0081732 | 178800656 | 0.721 | p<2.64E-07 | 6.05E-04 |
| 2 | H3GA0006772 | 50664844 | 0.721 | p<2.64E-07 | 6.72E-04 |
| 13 | ASGA0057460 | 50670413 | 0.721 | p<2.64E-07 | 6.72E-04 |
| 13 | MARC0048691 | 51720680 | 0.720 | p<2.64E-07 | 6.72E-04 |
| 14 | DIAS0002104 | 71887585 | 0.720 | p<2.64E-07 | 6.72E-04 |
| 14 | INRA0044651 | 71949322 | 0.720 | p<2.64E-07 | 6.72E-04 |
| 4 | INRA0014361 | 66945964 | 0.717 | p<2.64E-07 | 6.72E-04 |
| 7 | DRGA0007707 | 65801718 | 0.715 | p<2.64E-07 | 7.39E-04 |
| 1 | ASGA0003191 | 70701950 | 0.714 | p<2.64E-07 | 8.06E-04 |
| 14 | INRA0044654 | 71975247 | 0.714 | p<2.64E-07 | 8.73E-04 |
| 13 | ALGA0067679 | 6951525 | 0.710 | p<2.64E-07 | 8.73E-04 |
| 5 | DRGA0005921 | 63610034 | 0.710 | p<2.64E-07 | 9.41E-04 |
| 18 | ASGA0079081 | 18911413 | 0.710 | p<2.64E-07 | 9.41E-04 |
| 7 | H3GA0021745 | 56152593 | 0.708 | p<2.64E-07 | 1.01E-03 |
| 5 | ASGA0096617 | 80318867 | 0.705 | p<2.64E-07 | 1.07E-03 |
| 7 | INRA0026262 | 65984458 | 0.705 | p<2.64E-07 | 1.14E-03 |
| 14 | H3GA0040014 | 46325913 | 0.703 | p<2.64E-07 | 1.21E-03 |
| 8 | ALGA0048498 | 91350268 | 0.703 | p<2.64E-07 | 1.21E-03 |
| 7 | DRGA0007790 | 75365812 | 0.703 | p<2.64E-07 | 1.21E-03 |
| 8 | ALGA0048732 | 102433227 | 0.702 | p<2.64E-07 | 1.28E-03 |
| 14 | ALGA0077572 | 53170186 | 0.702 | p<2.64E-07 | 1.34E-03 |
| 14 | ASGA0063361 | 53182791 | 0.702 | p<2.64E-07 | 1.34E-03 |
| 14 | ASGA0063366 | 53237364 | 0.702 | p<2.64E-07 | 1.34E-03 |
| 11 | ALGA0060607 | 8143690 | 0.702 | p<2.64E-07 | 1.34E-03 |
| 13 | ASGA0090575 | 60165976 | 0.702 | p<2.64E-07 | 1.41E-03 |
| 17 | ASGA0078354 | 66811256 | 0.702 | p<2.64E-07 | 1.41E-03 |
| 14 | MARC0069902 | 71838418 | 0.697 | p<2.64E-07 | 1.41E-03 |
| 11 | MARC0060452 | 75523733 | 0.695 | p<2.64E-07 | 1.41E-03 |
| 7 | MARC0048797 | 90557027 | 0.694 | p<2.64E-07 | 1.41E-03 |
| 15 | ASGA0098805 | 44397220 | 0.691 | p<2.64E-07 | 1.48E-03 |
| 9 | ASGA0041227 | 7041512 | 0.690 | p<2.64E-07 | 1.48E-03 |
| 17 | ASGA0076049 | 32292040 | 0.689 | p<2.64E-07 | 1.48E-03 |
| 16 | ALGA0092291 | 82835496 | 0.688 | p<2.64E-07 | 1.48E-03 |
| 7 | ASGA0033712 | 53633798 | 0.688 | p<2.64E-07 | 1.48E-03 |
| 16 | ASGA0084351 | 30956011 | 0.687 | p<2.64E-07 | 1.48E-03 |
| 7 | M1GA0010676 | 105599606 | 0.686 | p<2.64E-07 | 1.48E-03 |
| 8 | MARC0103989 | 108700020 | 0.686 | p<2.64E-07 | 1.48E-03 |
| 8 | ALGA0048860 | 108759144 | 0.686 | p<2.64E-07 | 1.48E-03 |
| 8 | ASGA0039455 | 109102369 | 0.686 | p<2.64E-07 | 1.48E-03 |
| 9 | H3GA0026932 | 32210167 | 0.686 | p<2.64E-07 | 1.48E-03 |
| 13 | ALGA0069851 | 46650762 | 0.686 | p<2.64E-07 | 1.55E-03 |
| 14 | ASGA0064230 | 72927053 | 0.686 | p<2.64E-07 | 1.55E-03 |
| 1 | H3GA0003828 | 257175271 | 0.685 | p<2.64E-07 | 1.61E-03 |
| 11 | H3GA0031860 | 45537697 | 0.685 | p<2.64E-07 | 1.61E-03 |
| 11 | ALGA0060540 | 6139822 | 0.683 | p<2.64E-07 | 1.68E-03 |
| 1 | INRA0004030 | 134796719 | 0.682 | p<2.64E-07 | 1.68E-03 |
| 2 | ASGA0012369 | 143476881 | 0.682 | p<2.64E-07 | 1.68E-03 |
| 7 | DRGA0008069 | 110993314 | 0.682 | p<2.64E-07 | 1.68E-03 |
| 15 | H3GA0044550 | 86620801 | 0.681 | p<2.64E-07 | 1.75E-03 |
| 1 | ALGA0003901 | 70820192 | 0.681 | p<2.64E-07 | 1.75E-03 |
| 5 | ALGA0032384 | 66734344 | 0.681 | p<2.64E-07 | 1.75E-03 |
| 2 | ASGA0008845 | 7850065 | 0.680 | p<2.64E-07 | 1.75E-03 |
| 9 | ASGA0044136 | 116296807 | 0.679 | p<2.64E-07 | 1.75E-03 |
| 1 | MARC0023380 | 16221205 | 0.678 | p<2.64E-07 | 1.75E-03 |
| 9 | H3GA0028067 | 124298159 | 0.678 | p<2.64E-07 | 1.75E-03 |
| 7 | INRA0028522 | 121117779 | 0.678 | p<2.64E-07 | 1.75E-03 |
| 15 | MARC0018244 | 44862962 | 0.676 | p<2.64E-07 | 1.75E-03 |
| 11 | ALGA0061896 | 37109307 | 0.674 | p<2.64E-07 | 1.81E-03 |
| 10 | ASGA0046328 | 11135183 | 0.673 | p<2.64E-07 | 1.88E-03 |
| 9 | CASI0007446 | 96564969 | 0.672 | p<2.64E-07 | 1.95E-03 |
| 9 | DRGA0009731 | 123105472 | 0.671 | p<2.64E-07 | 2.02E-03 |
| 11 | ASGA0051206 | 68767837 | 0.671 | p<2.64E-07 | 2.08E-03 |
| 15 | INRA0059193 | 95919710 | 0.671 | p<2.64E-07 | 2.08E-03 |
| 1 | ALGA0006782 | 183316056 | 0.670 | p<2.64E-07 | 2.15E-03 |
| 9 | ASGA0041896 | 17607158 | 0.669 | p<2.64E-07 | 2.15E-03 |
| 9 | ASGA0043652 | 71603146 | 0.667 | p<2.64E-07 | 2.22E-03 |
| 7 | H3GA0021402 | 50260218 | 0.666 | p<2.64E-07 | 2.22E-03 |
| 14 | MARC0051922 | 59894427 | 0.665 | p<2.64E-07 | 2.22E-03 |
| 6 | ASGA0060337 | 138009749 | 0.663 | p<2.64E-07 | 2.22E-03 |
| 1 | ASGA0089933 | 281969962 | 0.663 | p<2.64E-07 | 2.22E-03 |
| 6 | H3GA0017536 | 9708474 | 0.662 | p<2.64E-07 | 2.22E-03 |
| 15 | ASGA0071822 | 157342833 | 0.661 | p<2.64E-07 | 2.22E-03 |
| 1 | CASI0003924 | 77862605 | 0.658 | p<2.64E-07 | 2.28E-03 |
| 13 | ALGA0071393 | 96252608 | 0.658 | p<2.64E-07 | 2.28E-03 |
| 9 | ALGA0106214 | 149004810 | 0.658 | p<2.64E-07 | 2.42E-03 |
| 11 | ASGA0049620 | 8555179 | 0.658 | p<2.64E-07 | 2.49E-03 |
| 10 | MARC0010149 | 66395187 | 0.657 | p<2.64E-07 | 2.49E-03 |
| 13 | ALGA0070645 | 70563982 | 0.655 | p<2.64E-07 | 2.49E-03 |
| 10 | ALGA0059426 | 61039457 | 0.655 | p<2.64E-07 | 2.49E-03 |
| 13 | H3GA0036408 | 52485722 | 0.654 | p<2.64E-07 | 2.55E-03 |
| 6 | ALGA0111913 | 74982947 | 0.654 | p<2.64E-07 | 2.55E-03 |
| 8 | H3GA0024419 | 18477511 | 0.652 | p<2.64E-07 | 2.55E-03 |
| 5 | ALGA0116381 | 68782481 | 0.650 | p<2.64E-07 | 2.62E-03 |
| 8 | ALGA0050287 | 146728917 | 0.650 | p<2.64E-07 | 2.62E-03 |
| 13 | MARC0072265 | 34775057 | 0.649 | p<2.64E-07 | 2.62E-03 |
| 1 | INRA0004170 | 143087067 | 0.648 | p<2.64E-07 | 2.62E-03 |
| 8 | MARC0066784 | 117423845 | 0.648 | p<2.64E-07 | 2.62E-03 |
| 7 | ALGA0037929 | 2410408 | 0.648 | p<2.64E-07 | 2.62E-03 |
| 13 | ALGA0068398 | 120191634 | 0.648 | p<2.64E-07 | 2.62E-03 |
| 4 | INRA0014994 | 82528375 | 0.648 | p<2.64E-07 | 2.69E-03 |
| 2 | DIAS0002757 | 121500017 | 0.647 | p<2.64E-07 | 2.69E-03 |
| 5 | DRGA0005851 | 55358266 | 0.647 | p<2.64E-07 | 2.69E-03 |
| 11 | ASGA0091145 | 73435073 | 0.647 | p<2.64E-07 | 2.69E-03 |
| 2 | ALGA0109755 | 154850978 | 0.646 | p<2.64E-07 | 2.75E-03 |
| 1 | ALGA0009657 | 288036346 | 0.646 | p<2.64E-07 | 2.82E-03 |
| 7 | MARC0005927 | 125942941 | 0.645 | p<2.64E-07 | 2.82E-03 |
| 7 | MARC0005928 | 125943001 | 0.645 | p<2.64E-07 | 2.82E-03 |
| 15 | ALGA0084063 | 17819850 | 0.645 | p<2.64E-07 | 2.96E-03 |
| 13 | DRGA0013434 | 211174368 | 0.645 | p<2.64E-07 | 3.02E-03 |
| 8 | ASGA0095368 | 145709748 | 0.644 | p<2.64E-07 | 3.02E-03 |
| 8 | ASGA0037967 | 17466843 | 0.644 | p<2.64E-07 | 3.09E-03 |
| 13 | H3GA0035602 | 16451575 | 0.644 | p<2.64E-07 | 3.16E-03 |
| 9 | DRGA0009483 | 82487047 | 0.643 | p<2.64E-07 | 3.16E-03 |
| 13 | H3GA0037600 | 173581438 | 0.643 | p<2.64E-07 | 3.16E-03 |
| 3 | ALGA0020145 | 100285031 | 0.643 | p<2.64E-07 | 3.22E-03 |
| 1 | ALGA0002350 | 34859535 | 0.643 | p<2.64E-07 | 3.29E-03 |
| 17 | ASGA0077752 | 60933924 | 0.643 | p<2.64E-07 | 3.29E-03 |
| 9 | ALGA0116806 | 145437597 | 0.642 | p<2.64E-07 | 3.29E-03 |
| 13 | ASGA0058242 | 84704827 | 0.642 | p<2.64E-07 | 3.29E-03 |
| 1 | ASGA0007965 | 303920214 | 0.641 | p<2.64E-07 | 3.29E-03 |
| 3 | ASGA0103961 | 32410365 | 0.641 | p<2.64E-07 | 3.29E-03 |
| 16 | M1GA0021378 | 82675301 | 0.641 | p<2.64E-07 | 3.29E-03 |
| 5 | MARC0076697 | 81316162 | 0.640 | p<2.64E-07 | 3.29E-03 |
| 16 | ASGA0073924 | 71748526 | 0.638 | p<2.64E-07 | 3.29E-03 |
| 16 | ALGA0090039 | 31983325 | 0.637 | p<2.64E-07 | 3.29E-03 |
| 4 | ALGA0025771 | 77786937 | 0.637 | p<2.64E-07 | 3.29E-03 |
| 13 | MARC0021871 | 60176849 | 0.636 | p<2.64E-07 | 3.29E-03 |
| 9 | ALGA0102832 | 21880289 | 0.636 | p<2.64E-07 | 3.29E-03 |
| 9 | MARC0114913 | 23412144 | 0.635 | p<2.64E-07 | 3.29E-03 |
| 17 | ASGA0075978 | 30466546 | 0.633 | p<2.64E-07 | 3.36E-03 |
| 1 | ALGA0008730 | 267994055 | 0.632 | p<2.64E-07 | 3.36E-03 |
| 1 | H3GA0004183 | 278639334 | 0.632 | p<2.64E-07 | 3.36E-03 |
| 13 | ASGA0058820 | 138941535 | 0.632 | p<2.64E-07 | 3.36E-03 |
| 2 | MARC0023931 | 20310468 | 0.632 | p<2.64E-07 | 3.36E-03 |
| 16 | DRGA0016097 | 36203828 | 0.631 | p<2.64E-07 | 3.36E-03 |
| 9 | ASGA0044847 | 140742311 | 0.631 | p<2.64E-07 | 3.36E-03 |
| 14 | ASGA0064202 | 72010321 | 0.631 | p<2.64E-07 | 3.43E-03 |
| 14 | ASGA0064204 | 72049542 | 0.631 | p<2.64E-07 | 3.43E-03 |
| 5 | ALGA0031967 | 55775955 | 0.630 | p<2.64E-07 | 3.43E-03 |
| 13 | MARC0019443 | 130982311 | 0.630 | p<2.64E-07 | 3.43E-03 |
| 14 | MARC0013889 | 72454386 | 0.630 | p<2.64E-07 | 3.43E-03 |
| 8 | DIAS0001596 | 32155082 | 0.630 | p<2.64E-07 | 3.49E-03 |
| 10 | H3GA0030664 | 68989822 | 0.630 | p<2.64E-07 | 3.49E-03 |
| 3 | ALGA0122760 | 79682219 | 0.629 | p<2.64E-07 | 3.56E-03 |
| 17 | MARC0070614 | 41305854 | 0.629 | p<2.64E-07 | 3.63E-03 |
| 4 | ASGA0019651 | 57775199 | 0.629 | p<2.64E-07 | 3.63E-03 |
| 2 | M1GA0002980 | 87338629 | 0.628 | p<2.64E-07 | 3.63E-03 |
| 3 | ASGA0014971 | 67809902 | 0.628 | p<2.64E-07 | 3.63E-03 |
| 5 | ALGA0032146 | 62612450 | 0.628 | p<2.64E-07 | 3.63E-03 |
| 9 | ASGA0042725 | 46912514 | 0.628 | p<2.64E-07 | 3.76E-03 |
| 11 | ALGA0061535 | 25305148 | 0.627 | p<2.64E-07 | 3.76E-03 |
| 13 | ALGA0070521 | 65899972 | 0.626 | p<2.64E-07 | 3.83E-03 |
| 14 | ALGA0075691 | 16504933 | 0.626 | p<2.64E-07 | 3.83E-03 |
| 14 | ASGA0061740 | 16541001 | 0.626 | p<2.64E-07 | 3.83E-03 |
| 15 | ALGA0119371 | 150369297 | 0.626 | p<2.64E-07 | 3.83E-03 |
| 6 | MARC0012805 | 109789591 | 0.625 | p<2.64E-07 | 3.83E-03 |
| 1 | INRA0005556 | 208268046 | 0.625 | p<2.64E-07 | 3.90E-03 |
| 7 | INRA0026374 | 71813234 | 0.625 | p<2.64E-07 | 3.90E-03 |
| 2 | M1GA0002493 | 6626294 | 0.625 | p<2.64E-07 | 3.90E-03 |
| 7 | ALGA0040661 | 40540791 | 0.625 | p<2.64E-07 | 3.90E-03 |
| 7 | INRA0026418 | 73938361 | 0.624 | p<2.64E-07 | 3.96E-03 |
| 7 | ASGA0034046 | 56230077 | 0.624 | p<2.64E-07 | 4.03E-03 |
| 9 | INRA0031367 | 21273498 | 0.624 | p<2.64E-07 | 4.03E-03 |
| 13 | ALGA0071057 | 85715978 | 0.624 | p<2.64E-07 | 4.17E-03 |
| 14 | MARC0057669 | 102962814 | 0.623 | p<2.64E-07 | 4.23E-03 |
| 1 | ALGA0004506 | 88602034 | 0.623 | p<2.64E-07 | 4.30E-03 |
| 16 | ASGA0099237 | 68468525 | 0.623 | p<2.64E-07 | 4.30E-03 |
| 3 | MARC0000971 | 128471642 | 0.623 | p<2.64E-07 | 4.37E-03 |
| 4 | ASGA0018712 | 17055319 | 0.623 | p<2.64E-07 | 4.37E-03 |
| 9 | ALGA0120617 | 2314907 | 0.623 | p<2.64E-07 | 4.37E-03 |
| 8 | ALGA0112756 | 138734873 | 0.623 | p<2.64E-07 | 4.37E-03 |
| 8 | ALGA0048786 | 105457647 | 0.622 | p<2.64E-07 | 4.37E-03 |
| 1 | MARC0042925 | 243257185 | 0.622 | p<2.64E-07 | 4.43E-03 |
| 1 | H3GA0001728 | 58761934 | 0.622 | p<2.64E-07 | 4.43E-03 |
| 1 | H3GA0003648 | 238169421 | 0.620 | p<2.64E-07 | 4.50E-03 |
| 13 | ALGA0070395 | 61211799 | 0.619 | p<2.64E-07 | 4.50E-03 |
| 15 | INRA0049525 | 75687558 | 0.619 | p<2.64E-07 | 4.57E-03 |
| 13 | H3GA0037549 | 166883259 | 0.619 | p<2.64E-07 | 4.57E-03 |
| 4 | INRA0014076 | 55206570 | 0.618 | p<2.64E-07 | 4.57E-03 |
| 3 | ALGA0108607 | 22584466 | 0.618 | p<2.64E-07 | 4.57E-03 |
| 13 | ASGA0057629 | 58672347 | 0.618 | p<2.64E-07 | 4.57E-03 |
| 11 | ASGA0049251 | 2719497 | 0.618 | p<2.64E-07 | 4.57E-03 |
| 1 | ALGA0004223 | 80788561 | 0.617 | p<2.64E-07 | 4.64E-03 |
| 12 | ALGA0064738 | 9053320 | 0.616 | p<2.64E-07 | 4.64E-03 |
| 10 | H3GA0029010 | 6616291 | 0.616 | p<2.64E-07 | 4.64E-03 |
| 13 | MARC0093666 | 79410199 | 0.615 | p<2.64E-07 | 4.70E-03 |
| 5 | M1GA0008129 | 99177734 | 0.613 | p<2.64E-07 | 4.70E-03 |
| 13 | DRGA0012449 | 60980258 | 0.613 | p<2.64E-07 | 4.70E-03 |
| 14 | MARC0009931 | 44862887 | 0.613 | p<2.64E-07 | 4.77E-03 |
| 15 | ASGA0069192 | 34365293 | 0.613 | p<2.64E-07 | 4.84E-03 |
| 9 | ASGA0098850 | 14706172 | 0.612 | p<2.64E-07 | 4.90E-03 |
| 1 | H3GA0003822 | 257096974 | 0.612 | p<2.64E-07 | 4.97E-03 |
| 13 | ASGA0060050 | 211816791 | 0.610 | p<2.64E-07 | 4.97E-03 |
| 12 | MARC0037889 | 32398409 | 0.610 | p<2.64E-07 | 5.04E-03 |
| 4 | ALGA0022568 | 6306405 | 0.608 | p<2.64E-07 | 5.04E-03 |
| 12 | MARC0049877 | 15536649 | 0.608 | p<2.64E-07 | 5.11E-03 |
| 4 | ALGA0023806 | 19867659 | 0.607 | p<2.64E-07 | 5.11E-03 |
| 8 | ASGA0040427 | 144569598 | 0.607 | p<2.64E-07 | 5.11E-03 |
| 12 | ALGA0066475 | 43261346 | 0.607 | p<2.64E-07 | 5.11E-03 |
| 16 | MARC0066925 | 31608126 | 0.607 | p<2.64E-07 | 5.17E-03 |
| 8 | ALGA0046848 | 21000928 | 0.606 | p<2.64E-07 | 5.17E-03 |
| 2 | ALGA0013993 | 54381571 | 0.606 | p<2.64E-07 | 5.24E-03 |
| 5 | ALGA0031618 | 34594796 | 0.605 | p<2.64E-07 | 5.24E-03 |
| 12 | ASGA0099886 | 59480153 | 0.605 | p<2.64E-07 | 5.31E-03 |
| 1 | ASGA0001464 | 44751939 | 0.605 | p<2.64E-07 | 5.37E-03 |
| 1 | DRGA0000613 | 42802898 | 0.604 | p<2.64E-07 | 5.37E-03 |
| 15 | ASGA0069923 | 87644991 | 0.604 | p<2.64E-07 | 5.44E-03 |
| 7 | ALGA0042559 | 78538720 | 0.604 | p<2.64E-07 | 5.44E-03 |
| 8 | H3GA0024897 | 61180840 | 0.604 | p<2.64E-07 | 5.51E-03 |
| 10 | ALGA0059770 | 67065984 | 0.603 | p<2.64E-07 | 5.58E-03 |
| 13 | ALGA0120107 | 42115333 | 0.603 | p<2.64E-07 | 5.64E-03 |
| 11 | INRA0036370 | 50386997 | 0.603 | p<2.64E-07 | 5.71E-03 |
| 11 | ALGA0106959 | 69162707 | 0.603 | p<2.64E-07 | 5.78E-03 |
| 10 | DIAS0001571 | 71545994 | 0.603 | p<2.64E-07 | 5.85E-03 |
| 9 | ALGA0123109 | 1343633 | 0.603 | p<2.64E-07 | 5.85E-03 |
| 7 | MARC0046128 | 41292382 | 0.603 | p<2.64E-07 | 5.91E-03 |
| 1 | H3GA0005283 | 305898703 | 0.602 | p<2.64E-07 | 5.98E-03 |
| 10 | ASGA0096701 | 67920836 | 0.602 | p<2.64E-07 | 5.98E-03 |
| 8 | ALGA0048745 | 103342939 | 0.602 | p<2.64E-07 | 6.05E-03 |
| 15 | ASGA0069921 | 87536020 | 0.602 | p<2.64E-07 | 6.11E-03 |
| 17 | ALGA0096314 | 65125689 | 0.602 | p<2.64E-07 | 6.11E-03 |
| 1 | M1GA0001437 | 284386253 | 0.602 | p<2.64E-07 | 6.11E-03 |
| 7 | MARC0089200 | 54356760 | 0.601 | p<2.64E-07 | 6.11E-03 |
| 13 | INRA0040972 | 134933329 | 0.601 | p<2.64E-07 | 6.11E-03 |
| 6 | ASGA0028486 | 67429179 | 0.599 | p<2.64E-07 | 6.11E-03 |
| 7 | ALGA0039950 | 31500144 | 0.599 | p<2.64E-07 | 6.11E-03 |
| 16 | H3GA0047277 | 80675264 | 0.599 | p<2.64E-07 | 6.11E-03 |
| 1 | ASGA0006016 | 257198514 | 0.598 | p<2.64E-07 | 6.25E-03 |
| 11 | H3GA0031293 | 10324482 | 0.598 | p<2.64E-07 | 6.25E-03 |
| 13 | ALGA0070440 | 63588824 | 0.598 | p<2.64E-07 | 6.25E-03 |
| 1 | MARC0086062 | 291219636 | 0.598 | p<2.64E-07 | 6.38E-03 |
| 14 | ASGA0103632 | 16303034 | 0.598 | p<2.64E-07 | 6.38E-03 |
| 6 | ALGA0124272 | 109856403 | 0.597 | p<2.64E-07 | 6.45E-03 |
| 1 | ALGA0007595 | 226458237 | 0.596 | p<2.64E-07 | 6.45E-03 |
| 3 | INRA0010950 | 83826850 | 0.596 | p<2.64E-07 | 6.52E-03 |
| 11 | MARC0090287 | 7229848 | 0.596 | p<2.64E-07 | 6.52E-03 |
| 15 | INRA0049834 | 95560399 | 0.595 | p<2.64E-07 | 6.52E-03 |
| 1 | ASGA0000050 | 1067148 | 0.595 | p<2.64E-07 | 6.58E-03 |
| 16 | ALGA0115696 | 83764692 | 0.595 | p<2.64E-07 | 6.58E-03 |
| 14 | ALGA0077711 | 58501164 | 0.594 | p<2.64E-07 | 6.65E-03 |
| 8 | ALGA0048742 | 102914965 | 0.594 | p<2.64E-07 | 6.65E-03 |
| 9 | ALGA0109785 | 140586919 | 0.593 | p<2.64E-07 | 6.65E-03 |
| 1 | DRGA0000615 | 42826453 | 0.592 | p<2.64E-07 | 6.65E-03 |
| 12 | MARC0033222 | 724868 | 0.592 | p<2.64E-07 | 6.65E-03 |
| 8 | M1GA0011804 | 11431939 | 0.591 | p<2.64E-07 | 6.79E-03 |
| 4 | ASGA0023491 | 142008163 | 0.591 | p<2.64E-07 | 6.79E-03 |
| 14 | ALGA0080831 | 117011153 | 0.591 | p<2.64E-07 | 6.79E-03 |
| 6 | ALGA0035645 | 73595056 | 0.591 | p<2.64E-07 | 6.79E-03 |
| 4 | ALGA0024380 | 33809902 | 0.590 | p<2.64E-07 | 6.85E-03 |
| 5 | DRGA0006105 | 86480453 | 0.590 | p<2.64E-07 | 6.85E-03 |
| 7 | H3GA0020462 | 27962187 | 0.590 | p<2.64E-07 | 6.92E-03 |
| 4 | DRGA0004480 | 15016104 | 0.590 | p<2.64E-07 | 6.92E-03 |
| 8 | ALGA0047919 | 57742090 | 0.589 | p<2.64E-07 | 6.92E-03 |
| 8 | ASGA0038830 | 57749812 | 0.589 | p<2.64E-07 | 6.92E-03 |
| 9 | MARC0019520 | 18740161 | 0.589 | p<2.64E-07 | 6.92E-03 |
| 8 | ALGA0048868 | 108949601 | 0.588 | p<2.64E-07 | 6.92E-03 |
| 14 | MARC0023129 | 130473311 | 0.588 | p<2.64E-07 | 6.92E-03 |
| 15 | ASGA0071241 | 143683671 | 0.588 | p<2.64E-07 | 6.99E-03 |
| 1 | H3GA0003232 | 184274507 | 0.588 | p<2.64E-07 | 7.05E-03 |
| 2 | ASGA0009797 | 29737228 | 0.588 | p<2.64E-07 | 7.05E-03 |
| 3 | MARC0004652 | 6555484 | 0.587 | p<2.64E-07 | 7.12E-03 |
| 3 | INRA0010926 | 81017085 | 0.586 | p<2.64E-07 | 7.12E-03 |
| 5 | ASGA0025490 | 46458445 | 0.586 | p<2.64E-07 | 7.19E-03 |
| 13 | ASGA0084805 | 49392505 | 0.586 | p<2.64E-07 | 7.26E-03 |
| 15 | MARC0011387 | 150236322 | 0.585 | p<2.64E-07 | 7.26E-03 |
| 17 | M1GA0022834 | 68150143 | 0.585 | p<2.64E-07 | 7.26E-03 |
| 4 | ALGA0023171 | 11921003 | 0.584 | p<2.64E-07 | 7.32E-03 |
| 7 | ASGA0037208 | 130031038 | 0.584 | p<2.64E-07 | 7.32E-03 |
| 9 | ASGA0043928 | 93206153 | 0.584 | p<2.64E-07 | 7.32E-03 |
| 11 | ASGA0049279 | 2874367 | 0.584 | p<2.64E-07 | 7.32E-03 |
| 13 | ALGA0069951 | 50692278 | 0.584 | p<2.64E-07 | 7.39E-03 |
| 15 | ALGA0086308 | 95120099 | 0.584 | p<2.64E-07 | 7.39E-03 |
| 16 | SIRI0000384 | 47011906 | 0.584 | p<2.64E-07 | 7.39E-03 |
| 1 | INRA0003187 | 95873800 | 0.583 | p<2.64E-07 | 7.39E-03 |
| 14 | ASGA0063417 | 56505090 | 0.583 | p<2.64E-07 | 7.46E-03 |
| 6 | MARC0033885 | 38728802 | 0.583 | p<2.64E-07 | 7.52E-03 |
| 6 | ASGA0099055 | 151199647 | 0.583 | p<2.64E-07 | 7.52E-03 |
| 8 | DRGA0009021 | 146645905 | 0.583 | p<2.64E-07 | 7.52E-03 |
| 2 | INRA0009829 | 137740731 | 0.583 | p<2.64E-07 | 7.59E-03 |
| 14 | ALGA0075891 | 19819996 | 0.583 | p<2.64E-07 | 7.59E-03 |
| 17 | ASGA0095765 | 8317267 | 0.583 | p<2.64E-07 | 7.59E-03 |
| 4 | INRA0015761 | 104773534 | 0.582 | p<2.64E-07 | 7.66E-03 |
| 16 | ALGA0091334 | 71462099 | 0.581 | p<2.64E-07 | 7.66E-03 |
| 16 | MARC0112445 | 68450924 | 0.581 | p<2.64E-07 | 7.66E-03 |
| 17 | MARC0020638 | 7116491 | 0.581 | p<2.64E-07 | 7.66E-03 |
| 4 | ALGA0024414 | 34261106 | 0.581 | p<2.64E-07 | 7.73E-03 |
| 10 | ALGA0058427 | 39507554 | 0.580 | p<2.64E-07 | 7.73E-03 |
| 1 | ASGA0006236 | 266400773 | 0.580 | p<2.64E-07 | 7.73E-03 |
| 7 | MARC0050901 | 127138266 | 0.579 | p<2.64E-07 | 7.73E-03 |
| 2 | ALGA0105204 | 152349639 | 0.579 | p<2.64E-07 | 7.79E-03 |
| 13 | MARC0014143 | 57724136 | 0.579 | p<2.64E-07 | 7.79E-03 |
| 16 | MARC0042233 | 71347260 | 0.579 | p<2.64E-07 | 7.79E-03 |
| 10 | MARC0078321 | 5502952 | 0.578 | p<2.64E-07 | 7.93E-03 |
| 16 | ALGA0091043 | 62739364 | 0.578 | p<2.64E-07 | 8.00E-03 |
| 5 | ALGA0031539 | 33869835 | 0.577 | p<2.64E-07 | 8.06E-03 |
| 9 | ASGA0042135 | 26841604 | 0.577 | p<2.64E-07 | 8.06E-03 |
| 14 | ALGA0078523 | 72327890 | 0.577 | p<2.64E-07 | 8.13E-03 |
| 14 | DRGA0014010 | 72383660 | 0.577 | p<2.64E-07 | 8.13E-03 |
| 7 | ASGA0033576 | 52305109 | 0.575 | p<2.64E-07 | 8.13E-03 |
| 2 | ASGA0012020 | 138103597 | 0.575 | p<2.64E-07 | 8.13E-03 |
| 3 | ASGA0016342 | 125262103 | 0.575 | p<2.64E-07 | 8.20E-03 |
| 3 | ALGA0021725 | 138053335 | 0.574 | p<2.64E-07 | 8.20E-03 |
| 13 | ALGA0071388 | 94830010 | 0.574 | p<2.64E-07 | 8.20E-03 |
| 2 | MARC0000488 | 87728463 | 0.573 | p<2.64E-07 | 8.33E-03 |
| 9 | MARC0107517 | 151645402 | 0.573 | p<2.64E-07 | 8.33E-03 |
| 10 | ASGA0047201 | 30592523 | 0.573 | p<2.64E-07 | 8.33E-03 |
| 2 | ISU10000081 | 140443704 | 0.572 | p<2.64E-07 | 8.47E-03 |
| 1 | ALGA0006655 | 179002367 | 0.571 | p<2.64E-07 | 8.47E-03 |
| 8 | CASI0010109 | 91211314 | 0.571 | p<2.64E-07 | 8.47E-03 |
| 6 | ALGA0036593 | 117787421 | 0.571 | p<2.64E-07 | 8.53E-03 |
| 1 | INRA0006961 | 278526937 | 0.570 | p<2.64E-07 | 8.60E-03 |
| 1 | ALGA0005829 | 138128675 | 0.570 | p<2.64E-07 | 8.67E-03 |
| 8 | ALGA0047052 | 27492570 | 0.569 | p<2.64E-07 | 8.67E-03 |
| 14 | ALGA0078623 | 75351353 | 0.569 | p<2.64E-07 | 8.67E-03 |
| 16 | DRGA0016027 | 30515922 | 0.568 | p<2.64E-07 | 8.80E-03 |
| 1 | ASGA0007552 | 297271814 | 0.567 | p<2.64E-07 | 8.87E-03 |
| 4 | ALGA0022893 | 8963064 | 0.567 | p<2.64E-07 | 8.87E-03 |
| 1 | ASGA0001011 | 13157349 | 0.567 | p<2.64E-07 | 8.87E-03 |
| 2 | ALGA0016105 | 138402066 | 0.567 | p<2.64E-07 | 8.94E-03 |
| 6 | MARC0075779 | 151865659 | 0.567 | p<2.64E-07 | 9.00E-03 |
| 13 | ASGA0057936 | 71661735 | 0.567 | p<2.64E-07 | 9.00E-03 |
| 4 | INRA0014318 | 65947562 | 0.566 | p<2.64E-07 | 9.07E-03 |
| 17 | ASGA0076841 | 43029792 | 0.566 | p<2.64E-07 | 9.07E-03 |
| 13 | ALGA0070735 | 73285195 | 0.566 | p<2.64E-07 | 9.14E-03 |
| 14 | INRA0044648 | 71804184 | 0.566 | p<2.64E-07 | 9.14E-03 |
| 9 | H3GA0026515 | 12026513 | 0.566 | p<2.64E-07 | 9.14E-03 |
| 9 | ASGA0044325 | 122776388 | 0.566 | p<2.64E-07 | 9.14E-03 |
| 15 | ASGA0071198 | 142979244 | 0.566 | p<2.64E-07 | 9.14E-03 |
| 13 | DRGA0013153 | 185763507 | 0.566 | p<2.64E-07 | 9.20E-03 |
| 16 | ASGA0072426 | 18897025 | 0.566 | p<2.64E-07 | 9.20E-03 |
| 8 | ALGA0049814 | 139688641 | 0.566 | p<2.64E-07 | 9.27E-03 |
| 2 | ALGA0013667 | 50130463 | 0.566 | p<2.64E-07 | 9.27E-03 |
| 9 | DIAS0002193 | 94841423 | 0.566 | p<2.64E-07 | 9.27E-03 |
| 1 | DIAS0002497 | 35373798 | 0.565 | p<2.64E-07 | 9.34E-03 |
| 9 | H3GA0054187 | 30292332 | 0.565 | p<2.64E-07 | 9.34E-03 |
| 11 | ASGA0049515 | 6160463 | 0.565 | p<2.64E-07 | 9.34E-03 |
| 1 | M1GA0000188 | 3189426 | 0.565 | p<2.64E-07 | 9.34E-03 |
| 1 | DIAS0003060 | 302939298 | 0.565 | p<2.64E-07 | 9.34E-03 |
| 2 | MARC0085978 | 44112723 | 0.565 | p<2.64E-07 | 9.34E-03 |
| 16 | M1GA0021006 | 47575422 | 0.565 | p<2.64E-07 | 9.34E-03 |
| 17 | DBMA0000205 | 20690224 | 0.565 | p<2.64E-07 | 9.34E-03 |
| 6 | MARC0102757 | 111892116 | 0.565 | p<2.64E-07 | 9.34E-03 |
| 15 | ALGA0084385 | 26598551 | 0.565 | p<2.64E-07 | 9.34E-03 |
| 14 | MARC0093587 | 37771899 | 0.565 | p<2.64E-07 | 9.41E-03 |
| 4 | MARC0090961 | 141582087 | 0.564 | p<2.64E-07 | 9.41E-03 |
| 4 | ALGA0025313 | 64780020 | 0.564 | p<2.64E-07 | 9.41E-03 |
| 18 | MARC0056600 | 38170145 | 0.564 | p<2.64E-07 | 9.41E-03 |
| 4 | INRA0014327 | 66089889 | 0.564 | p<2.64E-07 | 9.47E-03 |
| 14 | ALGA0076456 | 26717165 | 0.564 | p<2.64E-07 | 9.47E-03 |
| 9 | H3GA0028477 | 144236052 | 0.563 | p<2.64E-07 | 9.54E-03 |
| 13 | MARC0095474 | 83649050 | 0.563 | p<2.64E-07 | 9.54E-03 |
| 13 | ASGA0058396 | 89403686 | 0.563 | p<2.64E-07 | 9.61E-03 |
| 1 | ASGA0002324 | 40579200 | 0.563 | p<2.64E-07 | 9.61E-03 |
| 4 | ALGA0025367 | 67199718 | 0.563 | p<2.64E-07 | 9.61E-03 |
| 1 | ALGA0008753 | 268355367 | 0.563 | p<2.64E-07 | 9.67E-03 |
| 1 | ASGA0002159 | 35772766 | 0.563 | p<2.64E-07 | 9.67E-03 |
| 13 | MARC0033394 | 72574306 | 0.562 | p<2.64E-07 | 9.67E-03 |
| 3 | H3GA0056202 | 6558448 | 0.562 | p<2.64E-07 | 9.74E-03 |
| 1 | MARC0055938 | 267886675 | 0.562 | p<2.64E-07 | 9.74E-03 |
| 8 | ALGA0049288 | 124059371 | 0.561 | p<2.64E-07 | 9.74E-03 |
| 2 | ASGA0011029 | 106437552 | 0.560 | p<2.64E-07 | 9.74E-03 |
| 14 | H3GA0040774 | 72542700 | 0.560 | p<2.64E-07 | 9.74E-03 |
| 2 | M1GA0003510 | 157749166 | 0.560 | p<2.64E-07 | 9.74E-03 |
| 8 | ASGA0038241 | 27121327 | 0.560 | p<2.64E-07 | 9.74E-03 |
| 8 | ASGA0105040 | 105184824 | 0.559 | p<2.64E-07 | 9.81E-03 |
| 14 | ASGA0063037 | 44977107 | 0.559 | p<2.64E-07 | 9.81E-03 |
| 5 | ALGA0116249 | 487425 | 0.559 | p<2.64E-07 | 9.88E-03 |
| 1 | ALGA0113248 | 107825932 | 0.558 | p<2.64E-07 | 9.88E-03 |
| 1 | ALGA0005321 | 114653490 | 0.558 | p<2.64E-07 | 9.88E-03 |
| 6 | ALGA0037748 | 154145106 | 0.558 | p<2.64E-07 | 9.88E-03 |
| 13 | ALGA0069850 | 46618936 | 0.558 | p<2.64E-07 | 9.88E-03 |
| 10 | ALGA0118982 | 15659632 | 0.557 | p<2.64E-07 | 9.88E-03 |
| 6 | ALGA0124257 | 71042150 | 0.557 | p<2.64E-07 | 9.88E-03 |
| 13 | ASGA0090344 | 81743576 | 0.557 | p<2.64E-07 | 9.88E-03 |

Note: For comparison of TBP and WZSP, the Bonferroni corrected significant level (at α=0.01)=0.01/37,893 (# of SNPs analyzed)=2.64E-07.
