# Supplementary material for: Genomic Scan Reveals Loci under Altitude Adaptation in Tibetan and Dahe Pigs
Source: PLoS One. 2014 Oct 17;9(10):e110520. doi: 10.1371/journal.pone.0110520 (PMC4201535; doi:10.1371/journal.pone.0110520)
Supplement: Table S3 — List of significant SNPs in comparison of DHP and WZSP. PP , permutation p-value; PE , empirical p-value. (DOCX) [file pone.0110520.s003.docx]

**Table S3 List of significant SNPs in comparison of DHP and WZSP**

| **Chr** | **Name** | **Position** | **F_ST_** | ***P_P_*** | ***P_E_*** |
| --- | --- | --- | --- | --- | --- |
| 1 | INRA0006383 | 257072211 | 0.982 | p<3.06E-07 | 0.00E+00 |
| 16 | MARC0105115 | 30377127 | 0.982 | p<3.06E-07 | 0.00E+00 |
| 11 | ALGA0060607 | 8143690 | 0.965 | p<3.06E-07 | 7.73E-05 |
| 5 | MARC0076697 | 81316162 | 0.947 | p<3.06E-07 | 1.55E-04 |
| 6 | DRGA0006914 | 132505693 | 0.946 | p<3.06E-07 | 1.55E-04 |
| 5 | ALGA0032869 | 76055466 | 0.930 | p<3.06E-07 | 1.55E-04 |
| 1 | CASI0003924 | 77862605 | 0.912 | p<3.06E-07 | 2.32E-04 |
| 4 | M1GA0005888 | 57710354 | 0.912 | p<3.06E-07 | 2.32E-04 |
| 11 | ASGA0049454 | 5218722 | 0.912 | p<3.06E-07 | 2.32E-04 |
| 18 | MARC0089126 | 35689325 | 0.912 | p<3.06E-07 | 2.32E-04 |
| 7 | MARC0048797 | 90557027 | 0.910 | p<3.06E-07 | 3.09E-04 |
| 16 | DRGA0016027 | 30515922 | 0.909 | p<3.06E-07 | 3.86E-04 |
| 3 | MARC0045282 | 33605451 | 0.909 | p<3.06E-07 | 4.64E-04 |
| 13 | ASGA0090344 | 81743576 | 0.908 | p<3.06E-07 | 5.41E-04 |
| 2 | ASGA0012369 | 143476881 | 0.907 | p<3.06E-07 | 6.18E-04 |
| 5 | ALGA0116381 | 68782481 | 0.896 | p<3.06E-07 | 6.18E-04 |
| 15 | DRGA0015447 | 126154684 | 0.896 | p<3.06E-07 | 6.18E-04 |
| 16 | DRGA0016021 | 30264193 | 0.896 | p<3.06E-07 | 6.18E-04 |
| 1 | H3GA0055344 | 307006485 | 0.894 | p<3.06E-07 | 6.18E-04 |
| 7 | INRA0026262 | 65984458 | 0.894 | p<3.06E-07 | 6.18E-04 |
| 1 | ALGA0011137 | 311326633 | 0.893 | p<3.06E-07 | 6.96E-04 |
| 14 | ALGA0075891 | 19819996 | 0.893 | p<3.06E-07 | 6.96E-04 |
| 1 | H3GA0001331 | 34184694 | 0.891 | p<3.06E-07 | 7.73E-04 |
| 1 | DBUN0003645 | 92004937 | 0.891 | p<3.06E-07 | 7.73E-04 |
| 16 | H3GA0056734 | 82425544 | 0.891 | p<3.06E-07 | 7.73E-04 |
| 1 | ALGA0008336 | 256781800 | 0.890 | p<3.06E-07 | 8.50E-04 |
| 17 | MARC0083915 | 11482484 | 0.890 | p<3.06E-07 | 8.50E-04 |
| 14 | INRA0044416 | 65593467 | 0.890 | p<3.06E-07 | 8.50E-04 |
| 9 | ALGA0052095 | 29845383 | 0.889 | p<3.06E-07 | 8.50E-04 |
| 9 | H3GA0028067 | 124298159 | 0.887 | p<3.06E-07 | 8.50E-04 |
| 11 | H3GA0031860 | 45537697 | 0.882 | p<3.06E-07 | 8.50E-04 |
| 9 | ASGA0042725 | 46912514 | 0.879 | p<3.06E-07 | 9.28E-04 |
| 2 | DIAS0002725 | 134956526 | 0.878 | p<3.06E-07 | 9.28E-04 |
| 2 | ASGA0097814 | 50036228 | 0.876 | p<3.06E-07 | 9.28E-04 |
| 4 | ASGA0105383 | 100551612 | 0.876 | p<3.06E-07 | 9.28E-04 |
| 14 | H3GA0043496 | 151423065 | 0.876 | p<3.06E-07 | 9.28E-04 |
| 14 | ALGA0074699 | 7044169 | 0.875 | p<3.06E-07 | 1.00E-03 |
| 1 | H3GA0004183 | 278639334 | 0.875 | p<3.06E-07 | 1.00E-03 |
| 5 | MARC0051702 | 69691307 | 0.872 | p<3.06E-07 | 1.00E-03 |
| 6 | ASGA0084851 | 127956844 | 0.872 | p<3.06E-07 | 1.00E-03 |
| 16 | ASGA0072005 | 4744298 | 0.872 | p<3.06E-07 | 1.00E-03 |
| 1 | M1GA0000188 | 3189426 | 0.872 | p<3.06E-07 | 1.08E-03 |
| 4 | INRA0014076 | 55206570 | 0.872 | p<3.06E-07 | 1.08E-03 |
| 1 | DRGA0001126 | 77798848 | 0.870 | p<3.06E-07 | 1.08E-03 |
| 14 | ASGA0066770 | 136723909 | 0.870 | p<3.06E-07 | 1.16E-03 |
| 5 | ASGA0025407 | 36973186 | 0.870 | p<3.06E-07 | 1.16E-03 |
| 13 | MARC0072265 | 34775057 | 0.870 | p<3.06E-07 | 1.16E-03 |
| 3 | ASGA0015180 | 80136834 | 0.862 | p<3.06E-07 | 1.16E-03 |
| 13 | H3GA0035602 | 16451575 | 0.862 | p<3.06E-07 | 1.16E-03 |
| 14 | MARC0023129 | 130473311 | 0.862 | p<3.06E-07 | 1.16E-03 |
| 4 | ALGA0024689 | 41828274 | 0.858 | p<3.06E-07 | 1.31E-03 |
| 4 | MARC0096487 | 66120397 | 0.858 | p<3.06E-07 | 1.31E-03 |
| 9 | H3GA0028477 | 144236052 | 0.858 | p<3.06E-07 | 1.31E-03 |
| 15 | ASGA0093353 | 15507527 | 0.858 | p<3.06E-07 | 1.31E-03 |
| 11 | MARC0017738 | 30968349 | 0.857 | p<3.06E-07 | 1.47E-03 |
| 13 | ASGA0060090 | 212390419 | 0.854 | p<3.06E-07 | 1.55E-03 |
| 3 | MARC0023781 | 56005947 | 0.853 | p<3.06E-07 | 1.62E-03 |
| 4 | INRA0014374 | 67057104 | 0.851 | p<3.06E-07 | 1.62E-03 |
| 4 | ASGA0021815 | 117247420 | 0.851 | p<3.06E-07 | 1.62E-03 |
| 4 | ASGA0023491 | 142008163 | 0.851 | p<3.06E-07 | 1.62E-03 |
| 12 | ALGA0108818 | 49085493 | 0.851 | p<3.06E-07 | 1.62E-03 |
| 12 | ASGA0099886 | 59480153 | 0.851 | p<3.06E-07 | 1.62E-03 |
| 14 | INRA0046649 | 120391470 | 0.851 | p<3.06E-07 | 1.62E-03 |
| 16 | ASGA0104902 | 8294989 | 0.851 | p<3.06E-07 | 1.62E-03 |
| 1 | ALGA0004579 | 90916676 | 0.851 | p<3.06E-07 | 1.86E-03 |
| 5 | MARC0022126 | 93355629 | 0.851 | p<3.06E-07 | 1.86E-03 |
| 14 | MARC0095688 | 28552559 | 0.851 | p<3.06E-07 | 1.86E-03 |
| 5 | ALGA0032100 | 62217959 | 0.850 | p<3.06E-07 | 2.01E-03 |
| 9 | ALGA0112800 | 22728120 | 0.850 | p<3.06E-07 | 2.01E-03 |
| 16 | ASGA0074889 | 86875355 | 0.850 | p<3.06E-07 | 2.01E-03 |
| 15 | INRA0049250 | 53136583 | 0.849 | p<3.06E-07 | 2.01E-03 |
| 9 | ALGA0104102 | 143771757 | 0.845 | p<3.06E-07 | 2.01E-03 |
| 15 | ASGA0071198 | 142979244 | 0.845 | p<3.06E-07 | 2.01E-03 |
| 1 | ASGA0006012 | 257136966 | 0.844 | p<3.06E-07 | 2.01E-03 |
| 13 | ALGA0070711 | 73023057 | 0.844 | p<3.06E-07 | 2.01E-03 |
| 4 | ALGA0025541 | 71462915 | 0.840 | p<3.06E-07 | 2.01E-03 |
| 5 | ALGA0103533 | 84381656 | 0.840 | p<3.06E-07 | 2.01E-03 |
| 6 | ALGA0124037 | 63146455 | 0.840 | p<3.06E-07 | 2.01E-03 |
| 3 | ASGA0103768 | 16623368 | 0.839 | p<3.06E-07 | 2.09E-03 |
| 4 | INRA0013758 | 39830135 | 0.839 | p<3.06E-07 | 2.09E-03 |
| 12 | ASGA0087025 | 4632244 | 0.838 | p<3.06E-07 | 2.09E-03 |
| 6 | ALGA0117684 | 131254700 | 0.835 | p<3.06E-07 | 2.09E-03 |
| 11 | H3GA0031573 | 22753532 | 0.835 | p<3.06E-07 | 2.09E-03 |
| 1 | ALGA0010033 | 293514268 | 0.835 | p<3.06E-07 | 2.24E-03 |
| 10 | H3GA0029163 | 10995624 | 0.835 | p<3.06E-07 | 2.24E-03 |
| 17 | ALGA0095947 | 61024176 | 0.835 | p<3.06E-07 | 2.24E-03 |
| 1 | DRGA0001092 | 72094887 | 0.834 | p<3.06E-07 | 2.24E-03 |
| 4 | DRGA0004878 | 76868849 | 0.834 | p<3.06E-07 | 2.24E-03 |
| 1 | H3GA0001534 | 46139189 | 0.833 | p<3.06E-07 | 2.32E-03 |
| 3 | MARC0042215 | 50118060 | 0.832 | p<3.06E-07 | 2.40E-03 |
| 9 | ASGA0041993 | 21679405 | 0.832 | p<3.06E-07 | 2.40E-03 |
| 3 | MARC0052941 | 26537702 | 0.830 | p<3.06E-07 | 2.47E-03 |
| 6 | ASGA0099021 | 151199625 | 0.830 | p<3.06E-07 | 2.47E-03 |
| 16 | ALGA0090039 | 31983325 | 0.830 | p<3.06E-07 | 2.47E-03 |
| 17 | MARC0022795 | 66201398 | 0.830 | p<3.06E-07 | 2.47E-03 |
| 1 | ASGA0001637 | 25040176 | 0.830 | p<3.06E-07 | 2.55E-03 |
| 1 | H3GA0001700 | 57981015 | 0.830 | p<3.06E-07 | 2.55E-03 |
| 5 | ASGA0027245 | 108924282 | 0.830 | p<3.06E-07 | 2.55E-03 |
| 18 | ALGA0120578 | 6537450 | 0.830 | p<3.06E-07 | 2.78E-03 |
| 7 | DRGA0007790 | 75365812 | 0.828 | p<3.06E-07 | 2.78E-03 |
| 6 | M1GA0008617 | 65385149 | 0.827 | p<3.06E-07 | 2.86E-03 |
| 7 | ASGA0034100 | 56993159 | 0.827 | p<3.06E-07 | 2.86E-03 |
| 5 | H3GA0016659 | 69555670 | 0.822 | p<3.06E-07 | 2.86E-03 |
| 5 | MARC0005359 | 71817704 | 0.822 | p<3.06E-07 | 2.86E-03 |
| 6 | ASGA0029105 | 95355941 | 0.822 | p<3.06E-07 | 2.86E-03 |
| 13 | ASGA0059219 | 165873235 | 0.822 | p<3.06E-07 | 2.86E-03 |
| 12 | ALGA0107813 | 62837875 | 0.821 | p<3.06E-07 | 2.94E-03 |
| 2 | M1GA0002493 | 6626294 | 0.817 | p<3.06E-07 | 2.94E-03 |
| 6 | MARC0016324 | 59213073 | 0.817 | p<3.06E-07 | 2.94E-03 |
| 15 | ALGA0084063 | 17819850 | 0.817 | p<3.06E-07 | 2.94E-03 |
| 7 | ASGA0030938 | 7607697 | 0.817 | p<3.06E-07 | 3.01E-03 |
| 1 | INRA0001515 | 33720247 | 0.816 | p<3.06E-07 | 3.09E-03 |
| 7 | DIAS0002191 | 27841549 | 0.813 | p<3.06E-07 | 3.09E-03 |
| 13 | DRGA0012610 | 82496275 | 0.813 | p<3.06E-07 | 3.09E-03 |
| 17 | DIAS0000155 | 41587488 | 0.813 | p<3.06E-07 | 3.09E-03 |
| 17 | H3GA0049247 | 53856831 | 0.812 | p<3.06E-07 | 3.09E-03 |
| 15 | CASI0004262 | 131567339 | 0.811 | p<3.06E-07 | 3.09E-03 |
| 1 | SIRI0001487 | 299578776 | 0.811 | p<3.06E-07 | 3.17E-03 |
| 8 | ALGA0050287 | 146728917 | 0.811 | p<3.06E-07 | 3.17E-03 |
| 4 | DRGA0004858 | 75471802 | 0.810 | p<3.06E-07 | 3.17E-03 |
| 8 | ALGA0047251 | 30820808 | 0.810 | p<3.06E-07 | 3.17E-03 |
| 2 | ALGA0109062 | 157472208 | 0.809 | p<3.06E-07 | 3.25E-03 |
| 2 | ALGA0014601 | 101918372 | 0.809 | p<3.06E-07 | 3.25E-03 |
| 16 | H3GA0056580 | 83126778 | 0.809 | p<3.06E-07 | 3.25E-03 |
| 5 | ALGA0104151 | 91515597 | 0.807 | p<3.06E-07 | 3.32E-03 |
| 9 | ALGA0104523 | 7755887 | 0.804 | p<3.06E-07 | 3.32E-03 |
| 14 | ALGA0080554 | 111768740 | 0.804 | p<3.06E-07 | 3.32E-03 |
| 14 | ASGA0065999 | 118395858 | 0.803 | p<3.06E-07 | 3.32E-03 |
| 3 | ASGA0082138 | 3787182 | 0.803 | p<3.06E-07 | 3.32E-03 |
| 11 | H3GA0031079 | 4907917 | 0.803 | p<3.06E-07 | 3.32E-03 |
| 12 | MARC0072109 | 24202370 | 0.803 | p<3.06E-07 | 3.32E-03 |
| 1 | DRGA0001136 | 78501187 | 0.801 | p<3.06E-07 | 3.40E-03 |
| 8 | ALGA0048195 | 77348231 | 0.801 | p<3.06E-07 | 3.48E-03 |
| 16 | MARC0111433 | 1245081 | 0.801 | p<3.06E-07 | 3.48E-03 |
| 2 | ALGA0013993 | 54381571 | 0.799 | p<3.06E-07 | 3.56E-03 |
| 1 | DRGA0000977 | 65306294 | 0.798 | p<3.06E-07 | 3.56E-03 |
| 1 | ASGA0003091 | 65321144 | 0.798 | p<3.06E-07 | 3.56E-03 |
| 5 | ALGA0123811 | 84481290 | 0.798 | p<3.06E-07 | 3.71E-03 |
| 9 | ALGA0120864 | 22985623 | 0.798 | p<3.06E-07 | 3.71E-03 |
| 11 | MARC0059524 | 42642971 | 0.798 | p<3.06E-07 | 3.71E-03 |
| 13 | H3GA0037549 | 166883259 | 0.797 | p<3.06E-07 | 3.86E-03 |
| 17 | INRA0053015 | 23547083 | 0.796 | p<3.06E-07 | 3.86E-03 |
| 1 | ASGA0101182 | 41839164 | 0.795 | p<3.06E-07 | 3.94E-03 |
| 1 | H3GA0002156 | 91678763 | 0.794 | p<3.06E-07 | 3.94E-03 |
| 4 | INRA0014251 | 63316010 | 0.794 | p<3.06E-07 | 3.94E-03 |
| 4 | ASGA0021547 | 113151281 | 0.794 | p<3.06E-07 | 3.94E-03 |
| 6 | ALGA0123322 | 82756988 | 0.794 | p<3.06E-07 | 3.94E-03 |
| 7 | ALGA0041867 | 55365676 | 0.794 | p<3.06E-07 | 3.94E-03 |
| 4 | ALGA0024760 | 42953649 | 0.794 | p<3.06E-07 | 3.94E-03 |
| 8 | DRGA0008716 | 102205112 | 0.794 | p<3.06E-07 | 3.94E-03 |
| 14 | MARC0113902 | 36699210 | 0.794 | p<3.06E-07 | 3.94E-03 |
| 15 | INRA0049351 | 57530002 | 0.794 | p<3.06E-07 | 4.02E-03 |
| 5 | MARC0036317 | 69148871 | 0.794 | p<3.06E-07 | 4.02E-03 |
| 1 | ASGA0002324 | 40579200 | 0.794 | p<3.06E-07 | 4.02E-03 |
| 6 | ASGA0085124 | 60680828 | 0.794 | p<3.06E-07 | 4.02E-03 |
| 1 | H3GA0001966 | 78423848 | 0.791 | p<3.06E-07 | 4.17E-03 |
| 17 | ALGA0096558 | 67082195 | 0.791 | p<3.06E-07 | 4.17E-03 |
| 5 | H3GA0016849 | 76613694 | 0.791 | p<3.06E-07 | 4.25E-03 |
| 12 | ASGA0054400 | 39594525 | 0.789 | p<3.06E-07 | 4.25E-03 |
| 14 | H3GA0043253 | 148320504 | 0.789 | p<3.06E-07 | 4.25E-03 |
| 1 | ALGA0010673 | 303425035 | 0.789 | p<3.06E-07 | 4.33E-03 |
| 3 | ALGA0017827 | 15548974 | 0.789 | p<3.06E-07 | 4.33E-03 |
| 4 | MARC0004034 | 69764920 | 0.789 | p<3.06E-07 | 4.33E-03 |
| 13 | MARC0014143 | 57724136 | 0.789 | p<3.06E-07 | 4.48E-03 |
| 2 | H3GA0007162 | 92572915 | 0.789 | p<3.06E-07 | 4.56E-03 |
| 4 | ALGA0028500 | 127072671 | 0.789 | p<3.06E-07 | 4.56E-03 |
| 14 | H3GA0040656 | 66437563 | 0.789 | p<3.06E-07 | 4.56E-03 |
| 10 | ASGA0096596 | 44792079 | 0.787 | p<3.06E-07 | 4.56E-03 |
| 6 | ALGA0035952 | 85740131 | 0.787 | p<3.06E-07 | 4.64E-03 |
| 5 | INRA0019965 | 75579482 | 0.786 | p<3.06E-07 | 4.64E-03 |
| 5 | H3GA0016981 | 86773823 | 0.785 | p<3.06E-07 | 4.71E-03 |
| 9 | MARC0067620 | 18765671 | 0.785 | p<3.06E-07 | 4.71E-03 |
| 1 | ALGA0008338 | 256837213 | 0.785 | p<3.06E-07 | 4.71E-03 |
| 1 | ASGA0006006 | 256989077 | 0.785 | p<3.06E-07 | 4.71E-03 |
| 1 | DRGA0002145 | 257001258 | 0.785 | p<3.06E-07 | 4.71E-03 |
| 6 | ALGA0037605 | 148434913 | 0.785 | p<3.06E-07 | 4.71E-03 |
| 13 | ALGA0070557 | 66460974 | 0.785 | p<3.06E-07 | 4.71E-03 |
| 14 | DRGA0013748 | 28185433 | 0.785 | p<3.06E-07 | 4.71E-03 |
| 15 | ASGA0069398 | 42771206 | 0.783 | p<3.06E-07 | 4.79E-03 |
| 5 | H3GA0016978 | 86562093 | 0.781 | p<3.06E-07 | 4.79E-03 |
| 13 | DRGA0011984 | 11720192 | 0.780 | p<3.06E-07 | 4.79E-03 |
| 2 | DRGA0003586 | 135552272 | 0.779 | p<3.06E-07 | 4.79E-03 |
| 1 | ASGA0004378 | 132657598 | 0.779 | p<3.06E-07 | 4.79E-03 |
| 9 | H3GA0055384 | 15568758 | 0.778 | p<3.06E-07 | 4.79E-03 |
| 1 | ALGA0009857 | 291411312 | 0.778 | p<3.06E-07 | 4.87E-03 |
| 8 | ALGA0047923 | 58000986 | 0.778 | p<3.06E-07 | 4.87E-03 |
| 8 | MARC0040474 | 88066156 | 0.778 | p<3.06E-07 | 4.87E-03 |
| 5 | ASGA0026580 | 89130923 | 0.777 | p<3.06E-07 | 4.87E-03 |
| 5 | MARC0023942 | 67795920 | 0.777 | p<3.06E-07 | 4.95E-03 |
| 8 | H3GA0024679 | 31495164 | 0.777 | p<3.06E-07 | 4.95E-03 |
| 13 | INRA0040915 | 129358795 | 0.777 | p<3.06E-07 | 4.95E-03 |
| 1 | ASGA0007552 | 297271814 | 0.776 | p<3.06E-07 | 5.02E-03 |
| 8 | ALGA0113244 | 107954353 | 0.776 | p<3.06E-07 | 5.02E-03 |
| 9 | INRA0031367 | 21273498 | 0.776 | p<3.06E-07 | 5.02E-03 |
| 1 | MARC0004776 | 94259519 | 0.772 | p<3.06E-07 | 5.10E-03 |
| 16 | ASGA0074835 | 85619643 | 0.772 | p<3.06E-07 | 5.10E-03 |
| 5 | MARC0072196 | 84472955 | 0.771 | p<3.06E-07 | 5.18E-03 |
| 9 | ASGA0044768 | 138754600 | 0.771 | p<3.06E-07 | 5.18E-03 |
| 9 | DRGA0009832 | 138760952 | 0.771 | p<3.06E-07 | 5.18E-03 |
| 16 | ASGA0074743 | 82856026 | 0.770 | p<3.06E-07 | 5.18E-03 |
| 3 | ASGA0013843 | 21495074 | 0.770 | p<3.06E-07 | 5.18E-03 |
| 4 | ALGA0025367 | 67199718 | 0.770 | p<3.06E-07 | 5.18E-03 |
| 16 | ALGA0120801 | 29092396 | 0.770 | p<3.06E-07 | 5.18E-03 |
| 6 | ASGA0100505 | 5384951 | 0.769 | p<3.06E-07 | 5.26E-03 |
| 2 | ASGA0008845 | 7850065 | 0.769 | p<3.06E-07 | 5.26E-03 |
| 17 | ASGA0074908 | 225622 | 0.769 | p<3.06E-07 | 5.26E-03 |
| 1 | ASGA0001746 | 29539072 | 0.768 | p<3.06E-07 | 5.26E-03 |
| 5 | ALGA0032861 | 75815246 | 0.768 | p<3.06E-07 | 5.26E-03 |
| 9 | ALGA0118446 | 22965566 | 0.768 | p<3.06E-07 | 5.26E-03 |
| 4 | ALGA0116177 | 17652538 | 0.768 | p<3.06E-07 | 5.33E-03 |
| 6 | ALGA0120083 | 72172049 | 0.768 | p<3.06E-07 | 5.41E-03 |
| 7 | ALGA0043023 | 89255863 | 0.768 | p<3.06E-07 | 5.41E-03 |
| 9 | H3GA0027926 | 106558403 | 0.768 | p<3.06E-07 | 5.41E-03 |
| 1 | INRA0007576 | 297244594 | 0.768 | p<3.06E-07 | 5.49E-03 |
| 3 | MARC0016746 | 77446800 | 0.768 | p<3.06E-07 | 5.49E-03 |
| 9 | MARC0080160 | 133560503 | 0.768 | p<3.06E-07 | 5.49E-03 |
| 14 | MARC0084999 | 128345240 | 0.768 | p<3.06E-07 | 5.49E-03 |
| 4 | ALGA0026584 | 96701086 | 0.767 | p<3.06E-07 | 5.49E-03 |
| 9 | ASGA0041224 | 7123289 | 0.767 | p<3.06E-07 | 5.49E-03 |
| 14 | ASGA0093112 | 118807973 | 0.767 | p<3.06E-07 | 5.49E-03 |
| 15 | ASGA0071753 | 151968517 | 0.767 | p<3.06E-07 | 5.49E-03 |
| 3 | ASGA0103978 | 12226151 | 0.767 | p<3.06E-07 | 5.49E-03 |
| 17 | ASGA0076841 | 43029792 | 0.767 | p<3.06E-07 | 5.57E-03 |
| 1 | DRGA0001131 | 78300780 | 0.766 | p<3.06E-07 | 5.57E-03 |
| 17 | INRA0054998 | 67844394 | 0.766 | p<3.06E-07 | 5.57E-03 |
| 16 | MARC0108846 | 75360827 | 0.766 | p<3.06E-07 | 5.64E-03 |
| 6 | ASGA0095713 | 7643426 | 0.765 | p<3.06E-07 | 5.64E-03 |
| 17 | H3GA0049067 | 47468919 | 0.764 | p<3.06E-07 | 5.64E-03 |
| 8 | ALGA0048837 | 108117397 | 0.763 | p<3.06E-07 | 5.64E-03 |
| 2 | H3GA0007711 | 137241803 | 0.763 | p<3.06E-07 | 5.64E-03 |
| 9 | ASGA0043326 | 62996659 | 0.763 | p<3.06E-07 | 5.64E-03 |
| 13 | ALGA0070649 | 70647346 | 0.763 | p<3.06E-07 | 5.64E-03 |
| 1 | MARC0071323 | 245637785 | 0.762 | p<3.06E-07 | 5.64E-03 |
| 1 | ALGA0004176 | 78321306 | 0.761 | p<3.06E-07 | 5.64E-03 |
| 4 | ALGA0022941 | 9155394 | 0.761 | p<3.06E-07 | 5.64E-03 |
| 12 | MARC0115151 | 8422147 | 0.761 | p<3.06E-07 | 5.64E-03 |
| 5 | DIAS0001512 | 18588161 | 0.760 | p<3.06E-07 | 5.72E-03 |
| 9 | ALGA0054598 | 121058668 | 0.760 | p<3.06E-07 | 5.72E-03 |
| 13 | ALGA0104709 | 141658514 | 0.760 | p<3.06E-07 | 5.72E-03 |
| 3 | MARC0044793 | 59501141 | 0.760 | p<3.06E-07 | 5.80E-03 |
| 6 | ALGA0036881 | 126408320 | 0.760 | p<3.06E-07 | 5.80E-03 |
| 1 | INRA0003187 | 95873800 | 0.759 | p<3.06E-07 | 5.80E-03 |
| 10 | ASGA0100153 | 8345584 | 0.759 | p<3.06E-07 | 5.80E-03 |
| 15 | ASGA0092005 | 146298453 | 0.757 | p<3.06E-07 | 5.95E-03 |
| 4 | MARC0095887 | 126984212 | 0.756 | p<3.06E-07 | 5.95E-03 |
| 3 | ASGA0014928 | 66415731 | 0.755 | p<3.06E-07 | 6.03E-03 |
| 5 | ALGA0032715 | 71889421 | 0.755 | p<3.06E-07 | 6.03E-03 |
| 16 | ALGA0111237 | 83382500 | 0.755 | p<3.06E-07 | 6.03E-03 |
| 2 | MARC0073030 | 137193057 | 0.754 | p<3.06E-07 | 6.18E-03 |
| 4 | ALGA0025066 | 57837998 | 0.753 | p<3.06E-07 | 6.18E-03 |
| 16 | ASGA0093220 | 83806833 | 0.752 | p<3.06E-07 | 6.26E-03 |
| 15 | DRGA0015084 | 40592400 | 0.752 | p<3.06E-07 | 6.34E-03 |
| 7 | ALGA0041797 | 54761663 | 0.752 | p<3.06E-07 | 6.42E-03 |
| 13 | MARC0038928 | 38843344 | 0.751 | p<3.06E-07 | 6.49E-03 |
| 8 | ALGA0049814 | 139688641 | 0.751 | p<3.06E-07 | 6.57E-03 |
| 14 | INRA0043787 | 44548711 | 0.751 | p<3.06E-07 | 6.57E-03 |
| 4 | DIAS0000961 | 107282153 | 0.749 | p<3.06E-07 | 6.57E-03 |
| 9 | ASGA0042440 | 34993225 | 0.748 | p<3.06E-07 | 6.57E-03 |
| 9 | ALGA0053060 | 55850961 | 0.748 | p<3.06E-07 | 6.57E-03 |
| 14 | INRA0048622 | 153114220 | 0.748 | p<3.06E-07 | 6.57E-03 |
| 16 | ASGA0074687 | 82591060 | 0.748 | p<3.06E-07 | 6.65E-03 |
| 1 | ALGA0006655 | 179002367 | 0.748 | p<3.06E-07 | 6.65E-03 |
| 4 | INRA0014318 | 65947562 | 0.747 | p<3.06E-07 | 6.65E-03 |
| 4 | MARC0055526 | 86209504 | 0.747 | p<3.06E-07 | 6.65E-03 |
| 16 | ALGA0105347 | 76899833 | 0.747 | p<3.06E-07 | 6.65E-03 |
| 4 | ALGA0027662 | 114882426 | 0.747 | p<3.06E-07 | 6.88E-03 |
| 18 | MARC0020348 | 4056774 | 0.747 | p<3.06E-07 | 6.88E-03 |
| 7 | M1GA0009457 | 5194125 | 0.746 | p<3.06E-07 | 6.88E-03 |
| 1 | DRGA0001299 | 97584782 | 0.746 | p<3.06E-07 | 6.88E-03 |
| 9 | ALGA0050620 | 4479898 | 0.746 | p<3.06E-07 | 6.88E-03 |
| 9 | MARC0005452 | 136848852 | 0.746 | p<3.06E-07 | 6.88E-03 |
| 8 | MARC0074396 | 89821072 | 0.746 | p<3.06E-07 | 6.96E-03 |
| 15 | CASI0006652 | 12812847 | 0.744 | p<3.06E-07 | 6.96E-03 |
| 1 | DRGA0001287 | 96062622 | 0.744 | p<3.06E-07 | 7.03E-03 |
| 6 | ALGA0115562 | 68428074 | 0.744 | p<3.06E-07 | 7.03E-03 |
| 12 | ALGA0065337 | 16590416 | 0.744 | p<3.06E-07 | 7.03E-03 |
| 13 | ALGA0070735 | 73285195 | 0.744 | p<3.06E-07 | 7.03E-03 |
| 1 | INRA0003491 | 109874960 | 0.743 | p<3.06E-07 | 7.19E-03 |
| 8 | DIAS0001596 | 32155082 | 0.743 | p<3.06E-07 | 7.19E-03 |
| 3 | M1GA0004091 | 14862033 | 0.741 | p<3.06E-07 | 7.27E-03 |
| 5 | M1GA0008164 | 106046784 | 0.741 | p<3.06E-07 | 7.27E-03 |
| 9 | M1GA0012522 | 7069360 | 0.741 | p<3.06E-07 | 7.27E-03 |
| 4 | H3GA0012532 | 38655109 | 0.741 | p<3.06E-07 | 7.27E-03 |
| 11 | ASGA0050955 | 57349455 | 0.740 | p<3.06E-07 | 7.34E-03 |
| 8 | MARC0025408 | 120836105 | 0.734 | p<3.06E-07 | 7.42E-03 |
| 10 | MARC0091319 | 59082877 | 0.734 | p<3.06E-07 | 7.42E-03 |
| 7 | ASGA0031422 | 15946248 | 0.734 | p<3.06E-07 | 7.42E-03 |
| 10 | M1GA0013736 | 12462966 | 0.734 | p<3.06E-07 | 7.42E-03 |
| 1 | ASGA0002551 | 49171144 | 0.734 | p<3.06E-07 | 7.57E-03 |
| 7 | ALGA0042353 | 67107544 | 0.734 | p<3.06E-07 | 7.57E-03 |
| 13 | H3GA0038005 | 210838951 | 0.734 | p<3.06E-07 | 7.57E-03 |
| 4 | ALGA0025771 | 77786937 | 0.733 | p<3.06E-07 | 7.57E-03 |
| 6 | ASGA0090197 | 24704496 | 0.733 | p<3.06E-07 | 7.57E-03 |
| 8 | MARC0082463 | 34932995 | 0.733 | p<3.06E-07 | 7.57E-03 |
| 10 | ASGA0048596 | 64649136 | 0.732 | p<3.06E-07 | 7.65E-03 |
| 1 | ALGA0002730 | 41985796 | 0.732 | p<3.06E-07 | 7.73E-03 |
| 1 | ASGA0006516 | 278347457 | 0.731 | p<3.06E-07 | 7.73E-03 |
| 4 | INRA0014142 | 59949762 | 0.731 | p<3.06E-07 | 7.73E-03 |
| 5 | MARC0069758 | 68781544 | 0.731 | p<3.06E-07 | 7.73E-03 |
| 2 | DRGA0003377 | 120672815 | 0.731 | p<3.06E-07 | 7.81E-03 |
| 4 | ASGA0100718 | 17648393 | 0.731 | p<3.06E-07 | 7.81E-03 |
| 9 | ASGA0041896 | 17607158 | 0.730 | p<3.06E-07 | 7.96E-03 |
| 1 | H3GA0001153 | 28587229 | 0.730 | p<3.06E-07 | 8.04E-03 |
| 17 | ALGA0095654 | 57081074 | 0.730 | p<3.06E-07 | 8.04E-03 |
| 4 | ALGA0027223 | 107515240 | 0.730 | p<3.06E-07 | 8.04E-03 |
| 5 | ALGA0033221 | 86714839 | 0.730 | p<3.06E-07 | 8.04E-03 |
| 16 | ALGA0090354 | 38718781 | 0.730 | p<3.06E-07 | 8.04E-03 |
| 16 | ASGA0105458 | 59315997 | 0.730 | p<3.06E-07 | 8.04E-03 |
| 5 | DRGA0005640 | 34811624 | 0.730 | p<3.06E-07 | 8.19E-03 |
| 6 | DRGA0006901 | 131833539 | 0.730 | p<3.06E-07 | 8.19E-03 |
| 13 | ASGA0057936 | 71661735 | 0.729 | p<3.06E-07 | 8.35E-03 |
| 3 | ASGA0083590 | 52262671 | 0.729 | p<3.06E-07 | 8.42E-03 |
| 14 | ASGA0066452 | 132774835 | 0.729 | p<3.06E-07 | 8.42E-03 |
| 14 | ASGA0067411 | 144719642 | 0.729 | p<3.06E-07 | 8.42E-03 |
| 9 | ALGA0115323 | 28926573 | 0.728 | p<3.06E-07 | 8.66E-03 |
| 13 | MARC0060283 | 112268319 | 0.728 | p<3.06E-07 | 8.66E-03 |
| 8 | H3GA0024887 | 57780578 | 0.728 | p<3.06E-07 | 8.73E-03 |
| 8 | MARC0070843 | 57825713 | 0.728 | p<3.06E-07 | 8.73E-03 |
| 13 | MARC0021665 | 135177274 | 0.728 | p<3.06E-07 | 8.73E-03 |
| 12 | ASGA0053700 | 20949950 | 0.728 | p<3.06E-07 | 8.73E-03 |
| 9 | ALGA0054305 | 108348177 | 0.727 | p<3.06E-07 | 8.73E-03 |
| 11 | MARC0036277 | 70296691 | 0.727 | p<3.06E-07 | 8.73E-03 |
| 13 | MARC0015751 | 210652085 | 0.727 | p<3.06E-07 | 8.73E-03 |
| 2 | ALGA0109755 | 154850978 | 0.727 | p<3.06E-07 | 8.81E-03 |
| 5 | ASGA0025237 | 32661210 | 0.727 | p<3.06E-07 | 8.81E-03 |
| 8 | ASGA0089134 | 123141281 | 0.727 | p<3.06E-07 | 8.97E-03 |
| 8 | ALGA0115298 | 146937454 | 0.727 | p<3.06E-07 | 8.97E-03 |
| 15 | ASGA0070067 | 91479099 | 0.727 | p<3.06E-07 | 8.97E-03 |
| 16 | ASGA0071969 | 4216600 | 0.727 | p<3.06E-07 | 8.97E-03 |
| 1 | ALGA0109145 | 129268324 | 0.727 | p<3.06E-07 | 9.04E-03 |
| 2 | DRGA0003000 | 52042198 | 0.727 | p<3.06E-07 | 9.12E-03 |
| 14 | INRA0044304 | 62556977 | 0.727 | p<3.06E-07 | 9.12E-03 |
| 1 | H3GA0004187 | 278822935 | 0.726 | p<3.06E-07 | 9.12E-03 |
| 3 | H3GA0010846 | 129847043 | 0.726 | p<3.06E-07 | 9.20E-03 |
| 5 | MARC0076572 | 74415936 | 0.726 | p<3.06E-07 | 9.20E-03 |
| 9 | DRGA0009616 | 106489605 | 0.725 | p<3.06E-07 | 9.28E-03 |
| 16 | MARC0056874 | 32178981 | 0.725 | p<3.06E-07 | 9.28E-03 |
| 9 | DRGA0009451 | 77876687 | 0.725 | p<3.06E-07 | 9.35E-03 |
| 10 | MARC0083428 | 50418539 | 0.725 | p<3.06E-07 | 9.35E-03 |
| 1 | ALGA0008664 | 267132213 | 0.725 | p<3.06E-07 | 9.43E-03 |
| 3 | ASGA0014621 | 55803289 | 0.725 | p<3.06E-07 | 9.43E-03 |
| 15 | ALGA0119012 | 117641266 | 0.725 | p<3.06E-07 | 9.43E-03 |
| 8 | ALGA0048150 | 76311198 | 0.725 | p<3.06E-07 | 9.43E-03 |
| 3 | ALGA0017611 | 12093180 | 0.725 | p<3.06E-07 | 9.43E-03 |
| 5 | ASGA0098598 | 98025228 | 0.724 | p<3.06E-07 | 9.51E-03 |
| 14 | ALGA0081794 | 135296757 | 0.724 | p<3.06E-07 | 9.51E-03 |
| 14 | ASGA0064230 | 72927053 | 0.723 | p<3.06E-07 | 9.58E-03 |
| 1 | MARC0010520 | 157470492 | 0.722 | p<3.06E-07 | 9.66E-03 |
| 13 | MARC0069372 | 78780192 | 0.722 | p<3.06E-07 | 9.66E-03 |
| 9 | ASGA0084095 | 28414446 | 0.722 | p<3.06E-07 | 9.74E-03 |
| 4 | ALGA0026502 | 95292395 | 0.722 | p<3.06E-07 | 9.74E-03 |
| 5 | SIRI0000395 | 41151243 | 0.722 | p<3.06E-07 | 9.74E-03 |
| 5 | ASGA0026215 | 72400948 | 0.722 | p<3.06E-07 | 9.74E-03 |
| 1 | MARC0100716 | 216019228 | 0.720 | p<3.06E-07 | 9.82E-03 |
| 7 | DRGA0007824 | 78078157 | 0.720 | p<3.06E-07 | 9.82E-03 |
| 17 | ASGA0076738 | 41274645 | 0.720 | p<3.06E-07 | 9.82E-03 |
| 4 | ASGA0021036 | 102489326 | 0.720 | p<3.06E-07 | 9.82E-03 |
| 6 | H3GA0017528 | 8019390 | 0.720 | p<3.06E-07 | 9.82E-03 |
| 6 | MARC0022542 | 89956690 | 0.718 | p<3.06E-07 | 9.89E-03 |
| 15 | ALGA0107158 | 35759501 | 0.718 | p<3.06E-07 | 9.89E-03 |
| 1 | ASGA0101301 | 281777220 | 0.718 | p<3.06E-07 | 9.89E-03 |
| 15 | DRGA0015644 | 148085537 | 0.716 | p<3.06E-07 | 9.89E-03 |
| 10 | H3GA0030744 | 70551436 | 0.716 | p<3.06E-07 | 9.89E-03 |
| 6 | ALGA0115484 | 133181146 | 0.716 | p<3.06E-07 | 9.89E-03 |
| 7 | MARC0030656 | 98604384 | 0.716 | p<3.06E-07 | 9.97E-03 |

Note: For comparison of DHP and WZSP, the Bonferroni corrected significant level (at α=0.01)=0.01/32,729 (# of SNPs analyzed)= 3.06E-07.
